# Comprehensive analysis of the physiological and molecular responses of phosphate-solubilizing bacterium *Burkholderia gladioli* DJB4–8 in promoting maize growth

**DOI:** 10.3389/fpls.2025.1611674

**Published:** 2025-06-13

**Authors:** Dao-Jun Guo, Guo-Rong Yang, Pratiksha Singh, Juan-Juan Wang, Xue-Mei Lan, Rajesh Kumar Singh, Jing Guo, Yu-Die Dong, Dong-Ping Li, Bin Yang

**Affiliations:** ^1^ Key Laboratory of Hexi Corridor Resources Utilization of Gansu, Hexi University, Zhangye, Gansu, China; ^2^ College of Life Sciences and Engineering, Hexi University, Zhangye, Gansu, China; ^3^ Guangxi Key Laboratory of Sugarcane Genetic Improvement, Guangxi Academy of Agricultural Sciences, Nanning, Guangxi, China; ^4^ College of Life Sciences, Sichuan Agricultural University, Ya’an, Sichuan, China; ^5^ College of Agriculture and Ecological Engineering, Hexi University, Zhangye, Gansu, China

**Keywords:** *Burkholderia gladioli* DJB4-8, phosphate-solubilizing bacteria, PGP, maize, transcriptome, metabolomics

## Abstract

Phosphorus (P) is one of the essential macroelements for the growth of maize. The deficiency of P in maize will result in adverse effects, including chlorosis and reduced yield. The Hexi Corridor in China serves as the principal region for seed maize production, with chemical phosphate fertilizer remaining the predominant source of P delivery for local maize cultivation. Nonetheless, the agricultural non-point source pollution resulting from the prolonged application of artificial phosphate fertilizers is intensifying. P in farmland soil often exists in an insoluble form, which plants cannot directly absorb and utilize. Phosphate-solubilizing bacteria (PSB) in the rhizosphere are a kind of plant growth-promoting rhizobacteria (PGPR) that can transform insoluble P in soil into soluble P for plants to absorb and utilize. Utilizing PGPR in agricultural production is an ecological approach to achieving sustainable development in agricultural practices and output. In this study, 41 strains of bacteria were isolated from the rhizosphere soil of four maize varieties. According to an *in vitro* plant growth-promoting (PGP) feature study and 16S RNA molecular identification, *Burkholderia gladioli* DJB4-8, among all strains tested, exhibited the highest *in vitro* PGP activity, with a phosphate-solubilizing ability of 8.99 mg/L. By scanning electron microscope (SEM) and green fluorescent protein (GFP) labeling technique, it was found that strain DJB4–8 formed a colonization symbiotic system with maize roots. The inoculation of maize Zhengdan 958 with strain DJB4–8 altered the plant’s photosynthetic physiology and indole-3-acetic acid (IAA) level, and it also dramatically increased the plant’s growth rate. The combined analysis of transcriptome and metabolomics showed that the key genes and metabolites in the interaction between strain DJB4–8 and maize were mainly concentrated in plant growth key pathways such as plant hormone signal transduction, phenylalanine, tyrosine and tryptophan biosynthesis, phenylalanine metabolism, phenylpropane biosynthesis, pentose phosphate pathway, zeatin biosynthesis, amino sugar and nucleotide sugar metabolism, and glutathione metabolism. These findings shed light on the need for additional research into the mechanism of interaction between PSB and maize.

## Introduction

The Hexi Corridor in China is the principal site for maize production, with approximately one million acres dedicated to seed maize cultivation (Chen et al., 2023). P is essential for the growth and development of maize, mainly in promoting cell division and differentiation, affecting photosynthesis and nutrient absorption, promoting root development and flowering and fruiting, and affecting the yield and quality of maize (Lambers, 2022; Richardson et al., 2009; Sadiq et al., 2017). P in the soil frequently exists in insoluble forms, such as calcium phosphate, rendering it inaccessible for direct absorption and utilization by plants (Johnston and Richards, 2003; Deng and Dhar, 2023; Olsen et al., 1983). P deficiency has become a critical limiting factor affecting local seed maize production. Currently, maize’s primary source of P supply relies on chemical P fertilizers. Plant growth-promoting rhizobacteria (PGPR) are beneficial microorganisms that interact symbiotically with plants and positively affect plant growth (Bhattacharyya and Jha, 2012; Jha and Saraf, 2015; Parray et al., 2016). Phosphate-solubilizing bacteria (PSB) play a crucial role in the P cycle within the soil (Khan et al., 2009; Pande et al., 2017). PSB secrete abundant organic acids as metabolic byproducts and generate CO_2_ through respiratory processes, which react with water in the soil to form carbonic acid. These acidic compounds dissolve insoluble phosphate minerals (e.g., calcium phosphate and aluminum phosphate) in the soil, releasing plant-available phosphate ions for uptake and enhancing P bioavailability in agricultural systems (Chen et al., 2006; Prijambada et al., 2009; Billah et al., 2019; Prager and Cisneros-Rojas, 2017). Regarding organic P compounds, PSB can release enzymes like phosphatase and phytase (Zhang et al., 2014; Rawat et al., 2021). These enzymes break down organic P compounds, such as phospholipids, phytic acid, and nucleic acids, and release phosphate and carbohydrates for use by crops (Pan and Cai, 2023; Prabhu et al., 2019; Alori et al., 2017). PSB can satisfy maize’s P needs by creating a comparatively adequate phosphorus supply micro area surrounding the rhizosphere. It accelerates the use of P fertilizer and promotes maize growth and development (Ingle and Padole, 2017; Adnan et al., 2020). In addition to facilitating phosphorus activation in the soil, PSB also can enhance plant growth by modifying the type and quantity of root exudates, thereby augmenting the uptake of minerals such as potassium, calcium, manganese, iron, and zinc by plant roots (Yahya et al., 2021; Biswas et al., 2018). Additionally, PSB produce biocontrol chemicals that inhibit the growth of phytopathogens and lower the incidence of disease, such as cyanide, siderophores, lytic enzymes, and antibiotics. At the same time, they increase the surface area of plant roots by colonizing them, improving nutrient uptake efficiency (Mitra et al., 2020; Vassilev et al., 2006; Li et al., 2017). This collaborative association mitigates abiotic stress and rhizosphere acidification, enabling plants to acclimate to adverse environments while enhancing agricultural productivity and stress resilience (Dey et al., 2021; Vassilev et al., 2012).

Numerous soil types and biological settings are home to PSB, which include bacteria such as *Bacillus*, *Pseudomonas*, *Burkholderia*, *Erwinia*, *Agrobacterium*, *Serratia*, and *Flavobacterium*, and these microbes are essential to the cycling of soil P (Saeid et al., 2018; Soares et al., 2023; You et al., 2020). PSB has been found in the rhizosphere of economic crops such as soybeans, sweet potatoes, fruit trees, and vegetables, as well as cereal crops like wheat, maize, sugarcane, and rice (Shome et al., 2022; Marques et al., 2019; Wang et al., 2022; Sundara et al., 2002; Rahman et al., 2015). According to previous reports, *Pseudomonas fluorescens*, *Pseudomonas putida*, *Bacillus megaterium*, *Bacillus subtilis*, *Burkholderia cepacia*, *Serratia liquefaciens*, *Enterobacter cloacae*, *Arthrobacter* sp*haeroides*, *Arthrobacter globiformis*, and *Azotobacter chroococcum* are the main PSB found in the rhizosphere of maize (Vyas and Gulati, 2009; Ahmad et al., 2019; Zhao et al., 2014; Tang et al., 2020).

Leveraging the growth-promoting attributes of PSB to augment phosphorus uptake and use in maize, hence fostering maize development, represents an eco-friendly approach to reducing chemical fertilizer application in maize cultivation (Fathi, 2017). *Burkholderia* is a common rhizosphere bacterium with multifunctional growth-promoting potential, especially in maize and other gramineous crops (Gastélum et al., 2022; Elnahal et al., 2022). The combination of its phosphate-solubilizing ability and pleiotropic growth-promoting mechanism plays a vital role in improving the P utilization efficiency of maize, enhancing stress resistance, and promoting root development (Liu, 2021; Li et al., 2012). Particularly in low-phosphorus soil, *Burkholderia* sp. can raise the biomass by 15% to 30% and the amount of accessible P in the maize rhizosphere by 20% to 50%. For example, maize production increased by 22% in the tropical red soil experiment (Pal et al., 2022). According to certain research, inoculating maize plants with PSB boosted their P intake by 29.74% (Vincze et al., 2024). PSB may also further enhance the nutritional status of maize, facilitating the absorption of additional elements like potassium and nitrogen (Sekhar et al., 2020). The primary focus of research on PSB in the maize rhizosphere is to isolate strains and evaluate their ability to stimulate growth. However, the mechanism of interaction between these bacteria and maize is poorly understood. Employing omics technologies, particularly transcriptomics and metabolomics, it is possible to discover differentially expressed genes and metabolites, build networks of gene–metabolite interactions, and uncover the functions and connections of important genes and metabolites in the interaction process (Yang et al., 2021). The basic objectives of this study were to identify and screen PSB from the rhizosphere soil of maize seed production in China’s Hexi Corridor and i) investigate the plant growth-promoting (PGP) activity of the isolated PSB while conducting a phylogenetic analysis; ii) examine the morphological traits of the PSB strain *Burkholderia gladioli* DJB4–8 via scanning electron microscopy, development of green fluorescent protein (GFP) clone transformants of strain DJB4-8, and assessment of its colonization properties in maize; iii) assess agronomic characteristics, photosynthetic physiology, and biochemical enzyme activities of maize Zhengdan 958 following inoculation with strain DJB4-8; and iv) examine the interaction genes and metabolic variances between strain DJB4–8 and maize Zhengdan 958 utilizing transcriptomics and metabolomics. The results will provide a scientific basis for further study of the interaction mechanism between PSB and maize.

## Materials and methods

### Isolation of bacteria from rhizosphere soil of seed maize

Healthy seed maize plants were selected from various areas of China’s Hexi Corridor’s National Hybrid Seed Maize production base. After digging the maize roots, the loose soil was carefully shaken to remove it. A sterile brush was used to brush the soil 1–2 mm from the root surface. The soil samples were immediately taken to the lab in an ice box and kept at 4°C (less than 24 h). Four soil samples, each approximately 100 g, were collected. The collection of information for rhizosphere soil samples of seed maize is presented in **Supplementary Table S1**. One gram of rhizosphere soil was weighed and added to 9 mL of sterile normal saline (containing 0.05% Tween 80 for dispersion). Oscillates were vortexed for 2 min and allowed to stand for 30 s; the supernatant was taken as the stock solution (10^−1^ dilution) and then gradually diluted it to 10^−2^, 10^−3^, 10^−4^, 10^−5^, 10^−6^, and 10^−7^; 100 μL of each diluted soil solution was coated on the plate of PKO medium and cultured upside down at 32°C for 48 h. Subsequent to the growth of the colonies, the strains were purified based on the distinct colony morphologies. All strains were cultivated in two aliquots: one was supplemented with 20% glycerol and preserved at −80°C, while the other was maintained at 4°C.

### Testing of PGP characteristics of maize rhizosphere soil bacteria

The activity of dissolved inorganic phosphate in maize rhizosphere bacteria for plant growth promotion was measured using a specialized Pikovskaya’s agar culture plate; 5 µL of each test bacterial solution was placed in the middle of the plate. Each culture plate was sealed with a membrane and then placed flat in an incubator set at 32°C for 72 h prior to observation. The strain’s phosphate-solubilizing ability was qualitatively indicated by the presence or absence of a colorless translucent circle, and the diameter of the circle was positively correlated with the ability. PSB’s capacity to dissolve inorganic phosphate was quantitatively assessed using the molybdenum antimony colorimetric technique (Guo et al., 2023).

The colorimetric method of Nessler’s reagent was utilized to determine whether the strains can produce ammonia. Culture media were created by dividing them into 15-mL vials and adding 10 g of peptone and 5 g of sodium chloride per liter. After dividing each vial into 9.5 mL of sterilized culture media, 10 μL of each test bacterial solution was added. Every culture bottle was put in an oscillating incubator with a consistent temperature. After 48 h of cultivation at 32°C and 110 rpm, 0.5 mL of Nessler’s reagent was added and observed to see if the bacterial solution turned reddish brown instead of yellow. A change in hue indicates that the strain can produce ammonia, and the capacity to produce ammonia increases with color darkness.

The tested strains’ capacity to secrete siderophores was assessed using the chrome azurol S (CAS) agar plate method (Milagres et al., 1999). The center of the CAS culture plate was inoculated with 5 µL of each test bacterial solution. Before being observed, each culture plate was placed flat in an incubator set at 32°C for 72 h after sealing it with a sealing film. The strain’s capacity to secrete siderophores was shown by the presence or absence of the yellow halo, and the diameter of the yellow halo was positively connected with the strain’s siderophore strength.

Culture plates were prepared for qualitative examination by adding 1-aminocyclopropyl-1-carboxylic acid (ACC) to Dworkin and Foster (DF) media (Li et al., 2011). After inoculating DF–ACC culture plates with the bacterial solution, they were incubated for 72 h at 32°C. The size of the bacterial circle positively correlates with the strain’s capacity to use the ACC nitrogen source. The absence of a bacterial growth zone demonstrates the strain’s incapacity to utilize ACC as a nitrogen source.

### Identification and phylogenetic analysis of maize rhizosphere soil bacteria

The bacterial genomic DNA was extracted using a TIANamp Bacteria DNA Kit (TIANGEN Biotech Co., Ltd., Beijing, China) following the manufacturer’s instructions. Each bacterium underwent PCR amplification of its 16S rRNA sequences. The primers and reaction parameters of the PCR conditions are presented in **Supplementary Table S2**. After acquiring the PCR products, the SanPrep column DNA gel recovery kit (Shenggong Biotech Co., Shanghai, China) was employed. The kit’s directions are referred to as the unique operation method. Following gel recovery and purification, the PCR product was identified using a 1.2% agarose gel to guarantee that only one band was sequenced. Shanghai Biotech Co., Ltd., completed the sequencing of bacterial 16S rRNA sequences. To obtain a login number, the sequencing data were compared and uploaded to the official website of the National Center for Biotechnology Information (NCBI). Next, sequences were grouped using the Mega7.0 software to produce a PSB strain 16S rRNA gene phylogenetic tree.

### Colonization analysis of PSB in maize

Seedlings of the maize variety Zhengdan 958 were inoculated with strain DJB4–8 at a concentration of 1 × 10^6^ CFU mL^−1^ and grown for 3 days. Sterile scissors were used to cut samples of the maize roots and stems, and absorbent paper was used to absorb the surface moisture. After being promptly fixed for 2 h using an electron microscope fixative, the samples were moved to 4°C for storage. The fixed samples for testing were washed three times for 15 min each using 0.1 M phosphoric acid buffer (Pb; pH 7.4). The samples were soaked in alcohol solutions for 15 min before being transferred to isoamyl acetate for 15 min. The samples were sprayed with gold for 30 s using the ion sputtering machine (Hitachi MC1000, Tokyo, Japan). After that, a scanning electron microscope (SEM; HITACHI SU8100) was used to collect the images.

Guo’s approach (Guo et al., 2020) was used in three parent bindings to change strain DJB4-8’s GFP into DJB4-8/pPROBE-pTetr-TT-gfp. Using sterile water as a control, Zhengdan 958 maize seedlings were inoculated with a concentration of 1 × 10^6^ CFU mL^−1^ of the three parent conjugate transformant solutions. The seedlings were removed after 4 days of inoculation, and sterile water was used to wash the culture media and contaminants from the root surface. Using a fluorescence microscope, the maize roots were manually sliced to assess the colonization status of strain DJB4-8.

### Agronomic traits and physiological and biochemical evaluation of maize after inoculation

The pot specifications for the inoculation experiment had a height of 18 cm, a bottom diameter of 18 cm, and an upper diameter of 25 cm. Each pot contained approximately 8 kg of soil. The maize variety was Zhengdan 958, and the inoculation concentration of the bacterial solution was 1 × 10^6^ CFU mL^−1^. Maize seedling cultivation used seedling trays: the cultivation tray was 40 cm long and 30 cm wide, with 50 seedling holes per tray and one seedling per hole. The seedling substrate was sterilized with high-pressure steam. Maize seeds were soaked in 1% carbendazim for 0.5 h before being transplanted into seedling trays. Under conventional management in greenhouse conditions, after the maize grew to two to three– leaves, maize seedlings with consistent growth were selected for inoculation and transplantation. After removing the maize seedlings from the seedling tray, the root maize matrix was rinsed with sterile water, and the roots were allowed to soak in the test bacterial solution for approximately 1 h. They were transferred to the container as the control group, and then the maize seedlings’ roots were submerged in sterile water. Each of the 60 pots in the treatment and control groups had 30 pots and one maize seedling. Maize was merely irrigated and did not receive fertilizer while growing. The experimental facility was situated in Hexi University’s maize-growing base. The maize plants were carefully uprooted from the growth medium, adhering rhizospheric soil particles were gently removed through manual agitation, and the root system was thoroughly rinsed with deionized water and allowed to air-dry at ambient temperature under laboratory conditions. The root, stem, and leaf weights were measured separately. The experiment was repeated three times, with three maize plants taken from each repetition for testing.

The growth hormone indole-3-acetic acid (IAA) in maize leaves was measured using high-performance liquid chromatography (HPLC); 0.2 g aliquot of the maize roots was accurately weighed and subjected to cryogenic grinding in a liquid nitrogen-chilled mortar. The resulting powder was homogenized with a pre-cooled methanol–acidic solution (70%–80% v/v, 4°C) and maintained at 4°C for 12 h to facilitate extraction. Following homogenization, the mixture underwent centrifugation at 12,000 rpm for 10 min (4°C). The supernatant was then collected under controlled temperature conditions and designated as the primary extract (first solution). After centrifuging the residue, 0.5 mL of a 70%–80% methanol solution was added, mixed well, and extracted for 2 h at 4°C; the second solution (the supernatant) was centrifuged, and then the supernatant was mixed twice. The supernatant was evaporated to one-third of its volume at 4°C and lowered pressure, and then an equivalent volume of petroleum ether was added. After two or three repetitions of extraction and decolorization, triethylamine was added after static stratification, and the pH was lowered to 8.0. The supernatant was put in the cross-linked polyvinylpyrrolidone (CTFA) and allowed to sit at room temperature for 20 min at 150 rpm. After adjusting the pH to 3.0, the supernatant was removed from the centrifuge, extracted three times using ethyl acetate, and then evaporated at 40°C with reduced pressure until dry. Vortex oscillation was used to dissolve the mobile phase solution after adding it. Under the following chromatographic conditions, the sample was passed through a needle filter, and a WuFeng LC-100 HPLC was used to identify it: mobile phase A of 100% methanol and B of 0.1% acetic acid aqueous solution (A:B = 55:45), with an injection volume of 20 μL, a column temperature of 30°C, a column size of 150 mm × 4.6 mm × 5 μm, and a detection wavelength of 254 nm. The standard curve for detecting endogenous hormone IAA in plants is shown in **Supplementary Table S3**.

### Transcriptome analysis of maize inoculated with PSB

After 40 days of PSB inoculation, the maize root RNA was extracted using a kit (Omega Bio-Tek, Inc., Norcross, GA, US). For the RNA extraction procedure, the kit instructions were consulted. Since 1% agarose gel electrophoresis was used to detect RNA integrity, and NanoDrop 2000 was used to detect RNA purity and concentration, it was appropriate for the RNA standard of transcriptome sequencing (OD260/OD230 value greater than 2.0, OD260/OD280 value between 1.8 and 2.2, and Rin value greater than 6.5).

The RNA library was created using the TruSeq™ RNA sample preparation kit (Illumina, San Diego, CA, USA). The 200–300 bp cDNA enrichment fragment was recovered by 2% agarose gel electrophoresis. The sequencing platform utilizes Illumina NovaSeq 6000. The sequencing library quantitatively employs TBS380 (PicoGreen), and the sequencing read length was PE150. The specific sequencing of RNA-seq refers to the method proposed by Zhang et al. (2020). The processes of cDNA synthesis, library construction and sequencing, sequencing result calculation and assembly, screening of differentially expressed genes, and analysis of metabolic pathways followed those of Martin and Wang (2011). Using the maize inbred line B73 genome as a transcriptome annotation reference genome, the genes were analyzed and found to be significantly differentially expressed after screening using the Kyoto Encyclopedia of Genes and Genomes (KEGG) and Gene Ontology (GO). Utilizing the Goatools program (https://github.com/tanghaibao/GOatools), functional analysis was conducted on genes that exhibited substantial differential expression, and *p*-values were adjusted using the Sidak, Holm, Bonferroni, and false discovery procedures. *p* (pfdr) ≤ 0.05 is the *p*-value threshold for GO functional significance enrichment. KOBAS (http://kobas.cbi.pku.edu.cn/home.do) enriched the KEGG metabolic pathway. Several tests were run on the results using the Benjamini–Hochberg (BH) [false discovery rate (FDR)] method to control the false-positive rate.

For transcriptome validation, quantitative real-time PCR (qRT-PCR) was used to verify the accuracy of the data. Twelve significantly differentially expressed genes were randomly selected for qRT-PCR validation, with the glyceraldehyde-3-phosphate dehydrogenase gene (GAPDH) as an internal reference gene. The gene primer design software was Primer 5.0, and the sequence is presented in **Supplementary Table S4**. Using the remaining RNA from transcriptome sequencing, its quality was evaluated using NanoDrop 2000, and the RNA was reverse-transcribed into cDNA using the reverse transcription reagent (Takara, Dalian, China). The qRT-PCR instrument was LightCycler 480) II, and the relative gene expression level was determined using the 2^−ΔΔCt^ method (Fleige and Pfaffl, 2006).

### Metabolome analysis of maize inoculated with strain DJB4-8

The collected maize root samples were stored on ice, and the metabolites were extracted with 50% methanol buffer. Then, the supernatant was transferred to a new 96-hole plate for mass spectrometry analysis. In addition, an equal amount of 10 μL diluent from the extracted mixture of each sample was obtained and mixed evenly as the quality control sample. All samples were collected using the LC–MS system following the manufacturer’s instructions. An ultra-high-performance liquid chromatography (UPLC) system was used for chromatographic separation, and an Acquity UPLC T3 column was used for reversed-phase separation. The injection volume of each sample was 4 μL.

The metabolites eluted from the column were detected using a triple high-resolution tandem mass spectrometer 5600+. quadrupole-time of flight (Q-TOF) operates in positive ion and negative ion modes. The XCMS, CAMERA, and metaX tools in the R software (v3.5.2) were used to process the LC–MS raw data files after they were transformed into mzXML format. Combining m/z data with retention time (RT) allowed for the identification of each ion. The metabolites were annotated using the KEGG database, and the accuracy of metabolite identification was further verified using the internal metabolite fragment library. Peak intensity data underwent advanced preprocessing through Metax, followed by partial least squares–discriminant analysis (PLS-DA) to evaluate batch effects and identify outliers within the preprocessed dataset. Intergroup metabolite differences were statistically analyzed using Student’s t-test, with *p*-values adjusted via the Benjamini–Hochberg FDR correction method. Subsequently, supervised PLS-DA was implemented in Metax to quantify variable importance in projection (VIP) scores and discriminate group-specific differentiating factors. The VIP’s crucial value was set at 1.0 to select important metabolites.

Transcriptome and metabolome data were standardized through log2 transformation. The evaluation of correlation functions was based on the R language and normalized data Pearson’s correlation analysis between differentially expressed genes (DEGs) and differentially accumulated metabolites (DAMs). KEGG signaling pathway enrichment analysis was performed using DEGs and DAMs with Pearson’s correlation coefficient (PCC) ≥0.8. Then, the DEGs were mapped, and DAMs were grouped in the KEGG pathway diagram.

### Data and statistical analyses

All data were analyzed using the SPSS 22.0 software for one-way analysis of variance (ANOVA) and Student’s t-test. *p* < 0.05 means a significant difference, and *p* < 0.01 means a highly significant difference. The OmicStudio toolbox was used in bioinformatics analysis (https://www.omicstudio.cn/tool).

## Results

### Isolation and identification of bacteria from maize rhizosphere soil and their PGP activity

**Figure 1:**
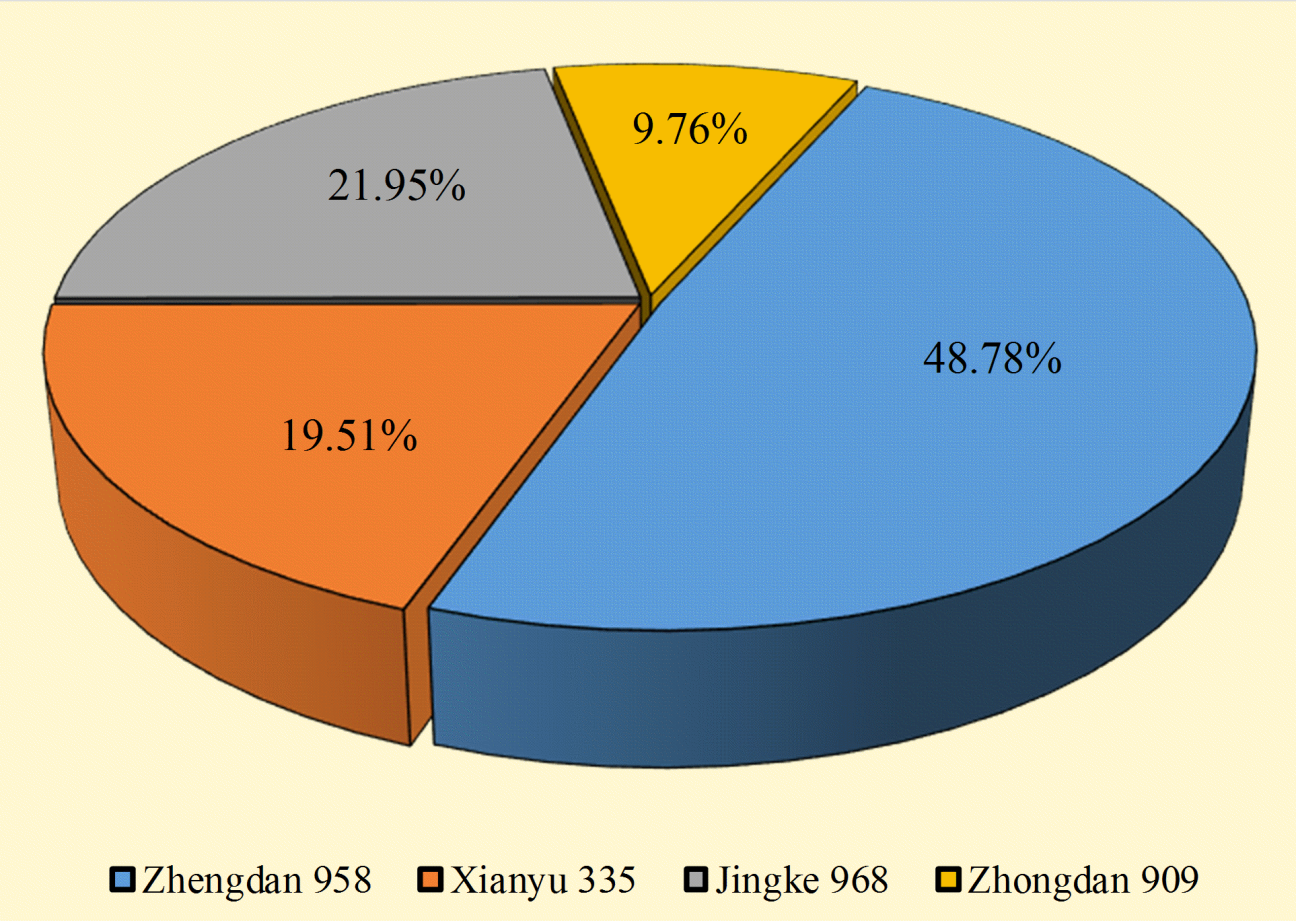
Isolation of bacterial strains from rhizosphere soil of four maize varieties.

**Figure 2:**
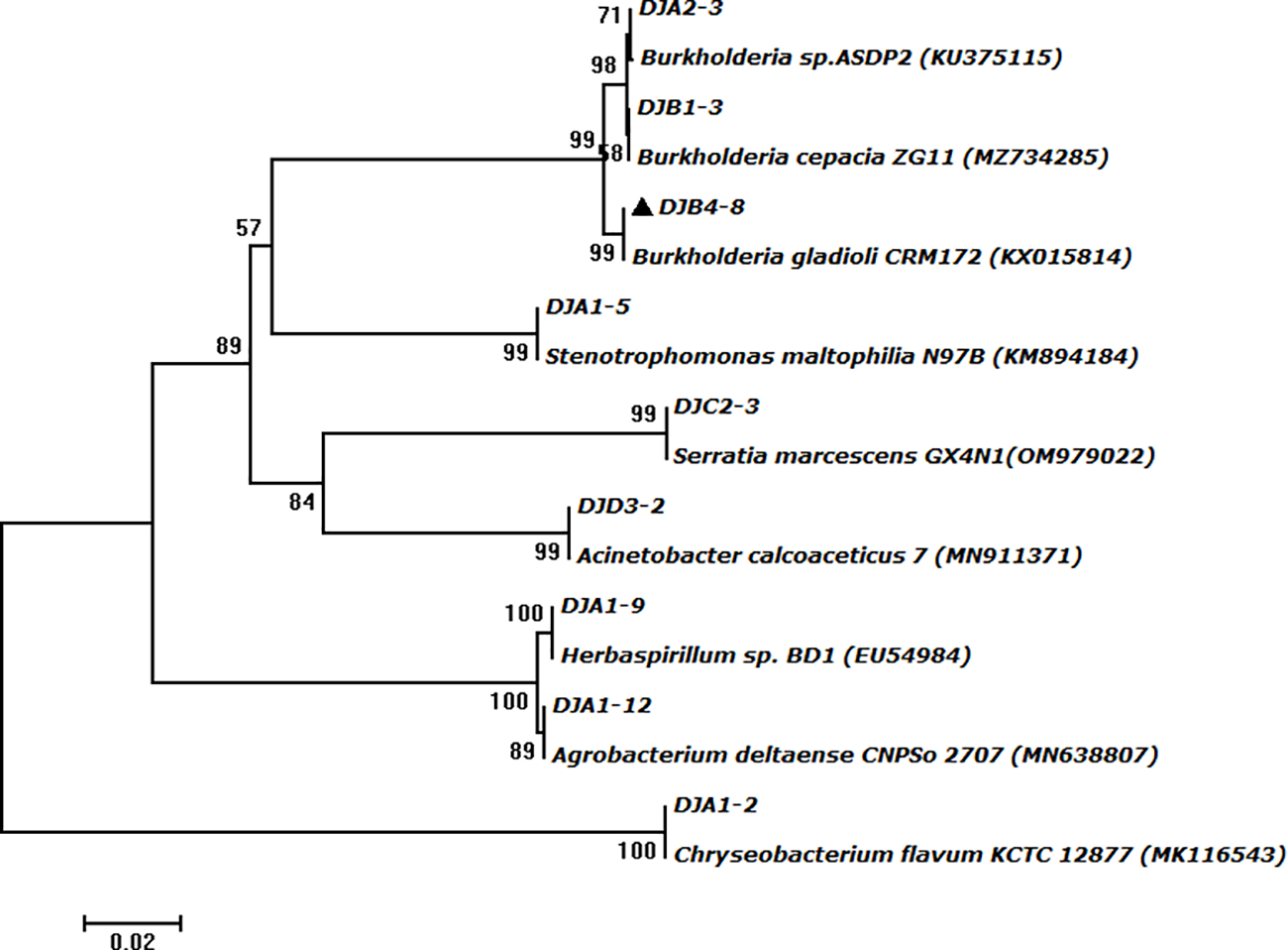
The phylogenetic tree was constructed based on the amplified 16S rRNA gene sequences of PSB strains from maize rhizosphere soil. PSB, phosphate-solubilizing bacteria.

A total of 41 strains of bacteria were isolated and purified from the rhizosphere soil of four seed maize varieties. Among them, 20, 8, 9, and 4 strains were isolated from maize varieties Zhengdan 958, Xianyu 335, Jingke 968, and Zhongdan 909, accounting for 48.78%, 19.51%, 21.95%, and 9.76%, respectively (**Figure 1**). Pikovskaya’s plate test found that nine strains of bacteria had strong phosphate-solubilizing ability, accounting for 21.95% of the total strains, in terms of their ability to dissolve inorganic phosphates (**Table 1**). Furthermore, 12 strains (29.26%) had vigorous ammonia-producing activity and the capacity to secrete siderophores (**Table 1**). However, only two strains had vigorous growth activity on the medium with ACC as the sole nitrogen source (**Table 1**). By analyzing the PGP characteristics of all strains, strain DJB4–8 showed intense activity in all detection indexes, which has the potential for further development. At the same time, the 16S rRNA identification of all strains showed that there were 12 genera of bacteria in 41 strains, of which *Burkholderia* was the dominant genus, with 17 strains (41.46%). Nine strains with strong inorganic phosphate-solubilizing ability were used to create a phylogenetic tree using the MEGA7.0 software, which showed that the strains DJB4–8 and *B. gladioli* had high similarities (**Figure 2**). The 16S rRNA sequences of all strains were submitted to the NCBI GenBank, and the strain accession number was PP792774-792814 (**Table 1**).

**Table 1:** Molecular identification and PGP characteristics of rhizosphere soil bacteria from four maize varieties.

Isolations	Most similar strain	Similarity (%)	NCBI accession no.	Phosphate	Ammonia	Siderophore	DF–ACC
DJA1-2	*Chryseobacterium flavum*	100	PP792774	+++	+++	−	++
DJA1-3	*Stenotrophomonas* sp.	99.93	PP792775	−	+	++	+
DJA1-5	*Stenotrophomonas calcoaceticus*	99	PP792776	+++	+	+	+
DJA1-9	*Herbaspirillum* sp.	99.93	PP792777	+++	++	−	++
DJA1-12	*Agrobacterium deltaense*	100	PP792778	+++	−	−	++
DJA2-2	*Enterobacter cloacae*	99.79	PP792779	++	+++	+	−
DJA2-3	*Burkholderia* sp.	99.93	PP792780	*+++*	*++*	*+*	++
DJA2-5	*Enterobacter* sp.	100	PP792781	++	+	++	−
DJA2-7	*Acinetobacter* sp.	100	PP792782	+	+	−	+
DJA2-8	*Burkholderia* sp.	99.93	PP792783	++	++	+++	+
DJA2-9	*Agrobacterium* sp.	99.85	PP792784	−	+	−	+
DJA3-2	*Microbacterium* sp.	99.93	PP792785	−	+	−	+
DJA3-4	*Herbaspirillum seropedicae*	100	PP792786	+	++	++	+
DJA3-5	*B. gladioli*	100	PP792787	++	++	++	+
DJA4-2	*Enterobacter cancerogenus*	99.64	PP792788	++	++	−	+
DJA4-3	*Pantoea dispersa*	99.93	PP792789	++	++	+++	+
DJA4-5	*Herbaspirillum huttiense*	99.93	PP792790	+	+	+	+
DJA4-6	*B. gladioli*	100	PP792791	++	+++	+++	+
DJA4-9	*Enterobacter ludwigii*	99.86	PP792792	++	+++	+	+
DJA5-3	*Herbaspirillum* sp.	100	PP792793	++	+	−	++
DJB1-3	*Burkholderia* sp.	99.86	PP792794	+++	++	+++	+++
DJB1-4	*Burkholderia* sp.	99.93	PP792795	++	++	+	++
DJB1-6	*Burkholderia cenocepacia*	99.71	PP792796	++	+++	+++	++
DJB2-3	*B. cenocepacia*	99.64	PP792797	++	+++	+++	+
DJB2-8	*B. gladioli*	99.93	PP792798	++	+++	+	+
DJB4-5	*B. cenocepacia*	100	PP792799	++	+++	+++	++
DJB4-8	*B. gladioli*	99.93	PP792800	+++	+++	+++	+++
DJB5-1	*Burkholderia* sp.	99.45	PP792801	++	++	+++	+
DJC1-3	*Pantoea ananatis*	100	PP792802	++	++	+	+
DJC1-4	*Stenotrophomonas* sp.	99.93	PP792803	++	+	+	+
DJC2-3	*Serratia marcescens*	100	PP792804	+++	++	++	++
DJC3-1	*Herbaspirillum* sp.	99.93	PP792805	+	+	++	++
DJC3-2	*B. cenocepacia*	100	PP792806	+	+	+	+
DJC3-4	*Burkholderia* sp.	99.79	PP792807	++	+++	+++	+
DJC4-2	*Burkholderia* sp.	99.86	PP792808	++	+	+++	+
DJC4-3	*P. ananatis*	99.72	PP792809	++	+	−	+
DJC4-8	*Burkholderia* sp.	99.71	PP792810	−	+++	+++	+
DJD1-1	*Sphingomonas* sp.	99.86	PP792811	++	++	−	+
DJD3-2	*Acinetobacter calcoaceticus*	100	PP792812	+++	++	++	+
DJD4-1	*Acidovorax avenae*	99.93	PP792813	++	+	−	+
DJD4-6	*Burkholderia cepacia*	99.86	PP792814	++	+++	++	+

The symbols “+++”, “++”, “+”, and “−” correspond to strong, moderate, weak, and undetectable levels of PGP activity, respectively, as demonstrated by the bacterial strains under standardized assay conditions.

PGP, plant growth-promoting; NCBI, National Center for Biotechnology Information; ACC, 1-aminocyclopropyl-1-carboxylic acid.

### Quantitative analysis of the ability of bacterial strains to dissolve inorganic phosphates

**Figure 3:**
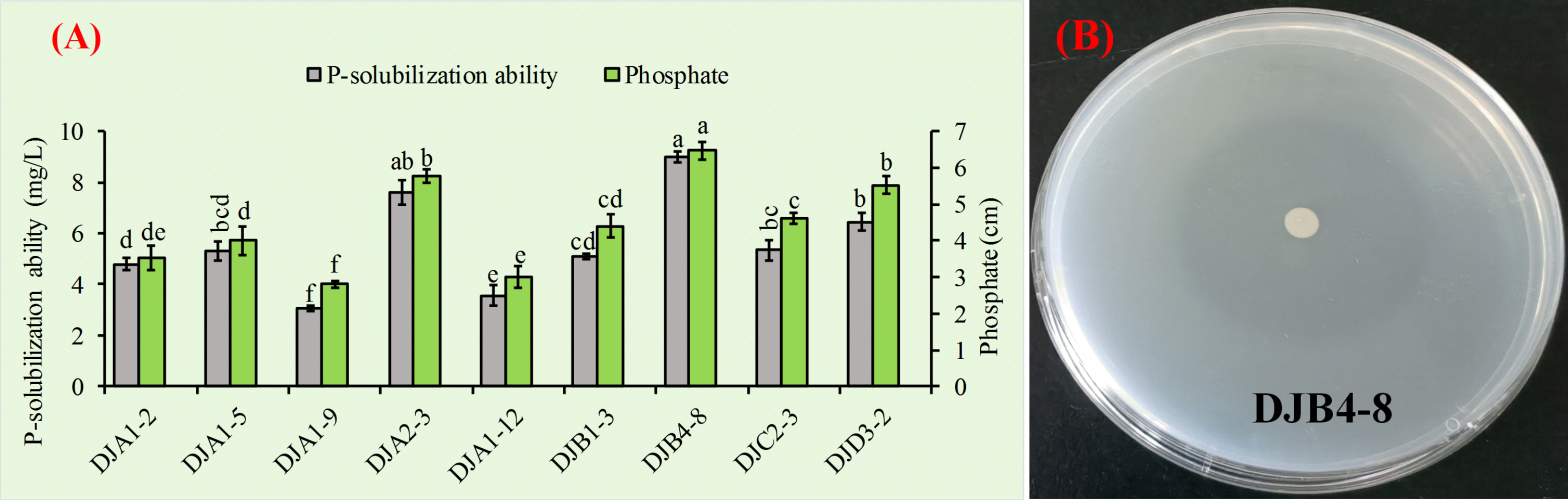
Assessment of phosphate-solubilizing capacity in PSB strains. **(A)** Quantitative determination of inorganic phosphate dissolution using the molybdenum-blue spectrophotometric method, demonstrating strain-specific solubilization efficiency. **(B)** Characterization of phosphate-releasing activity in the PSB strain DB4–8 through halo formation analysis on Pikovskaya’s agar medium. PSB, phosphate-solubilizing bacteria. Different letters on the column indicate significant difference at 0.05 level. Student T-test was used for the determination of significant differences.

The molybdenum antimony colorimetric method was used to quantitatively analyze the dissolution ability of nine strains that were proven to have a strong ability to dissolve inorganic phosphate. The results showed that PSB had a range of 3.05 to 8.99 mg/L for this ability (**Figure 3A**). Among them, strain DJA1–9 had the lowest quantitative ability to dissolve inorganic phosphate, while strain DJB4–8 had the highest. Notably, strain DJB4–8 demonstrated the maximum phosphate solubilization capacity among tested isolates, with a halo zone diameter of 6.47 cm (*p* < 0.01 vs. controls), as quantified in **Figure 3B**. Strain DJB4–8 shows excellent potential for further research and use.

### Colonization analysis of strain *B. gladioli* DJB4–8 in maize roots

**Figure 4:**
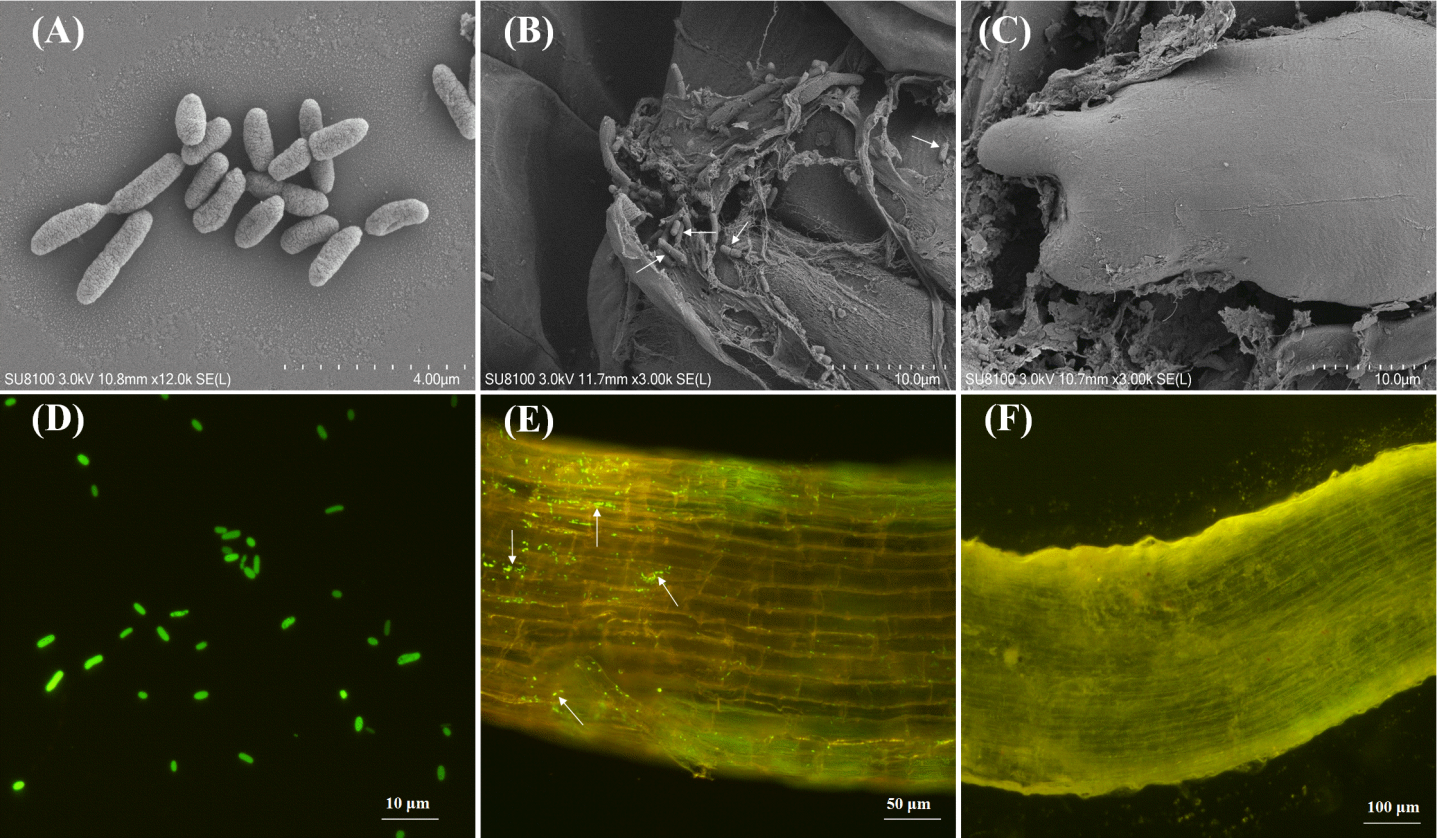
Using microscopic techniques to visualize DJB4–8 strain colonization dynamics in Zhengdan 958 maize root system. **(A)** SEM characterization of DJB4–8 colonial morphology. **(B)** SEM micrograph demonstrating successful rhizoplane colonization by DJB4–8 on Zhengdan 958 maize roots. **(C)** Comparative SEM analysis of root architecture in non-inoculated control plants. **(D)** Epifluorescence microscopic detection of GFP-expressing DJB4–8 colonies. **(E)** Spatial distribution pattern of GFP-tagged DJB4–8 colonizing Zhengdan 958 root tissues revealed by fluorescence microscopy. **(F)** Autofluorescence control imaging of uninoculated Zhengdan 958 root specimens. GFP, green fluorescent protein.

The SEM imaging revealed the successful rhizospheric colonization of maize roots by strain DJB4-8 (**Figures 4A–C**). Complementary to these morphological observations, the GFP-tagged DJB4–8 derivatives demonstrated sustained endophytic colonization patterns within root cortical tissues by fluorescence microscopy (**Figures 4D–F**). This multimodal imaging evidence collectively confirms the establishment of functional symbiotic associations between DJB4–8 and *Zea mays* host plants.

### Effect analysis of maize inoculated with strain *B. gladioli* DJB4-8

**Figure 5:**
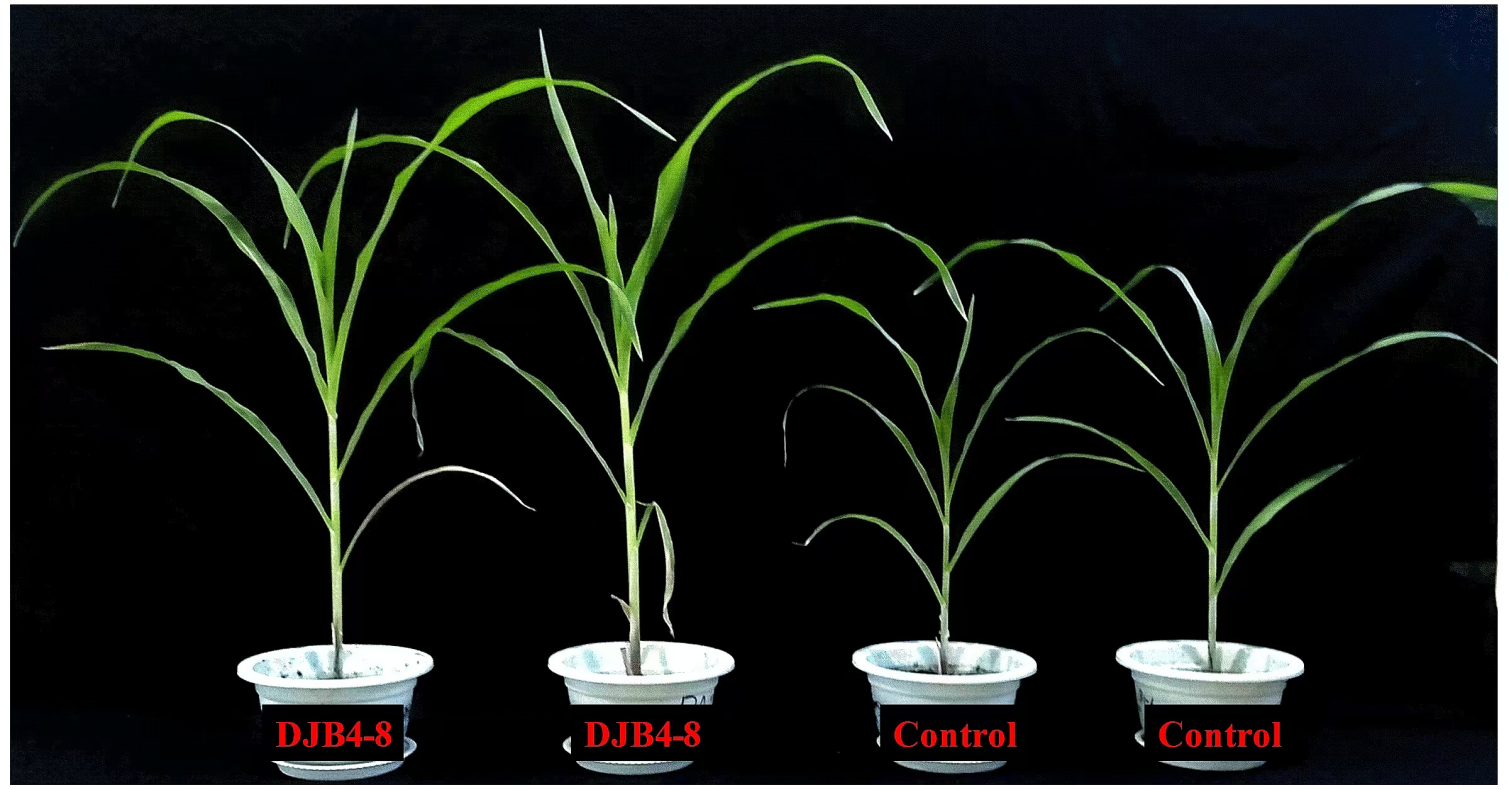
Growth status of strain DJB4–8 inoculated with maize Zhengdan 958 at 40 days.

**Figure 6:**
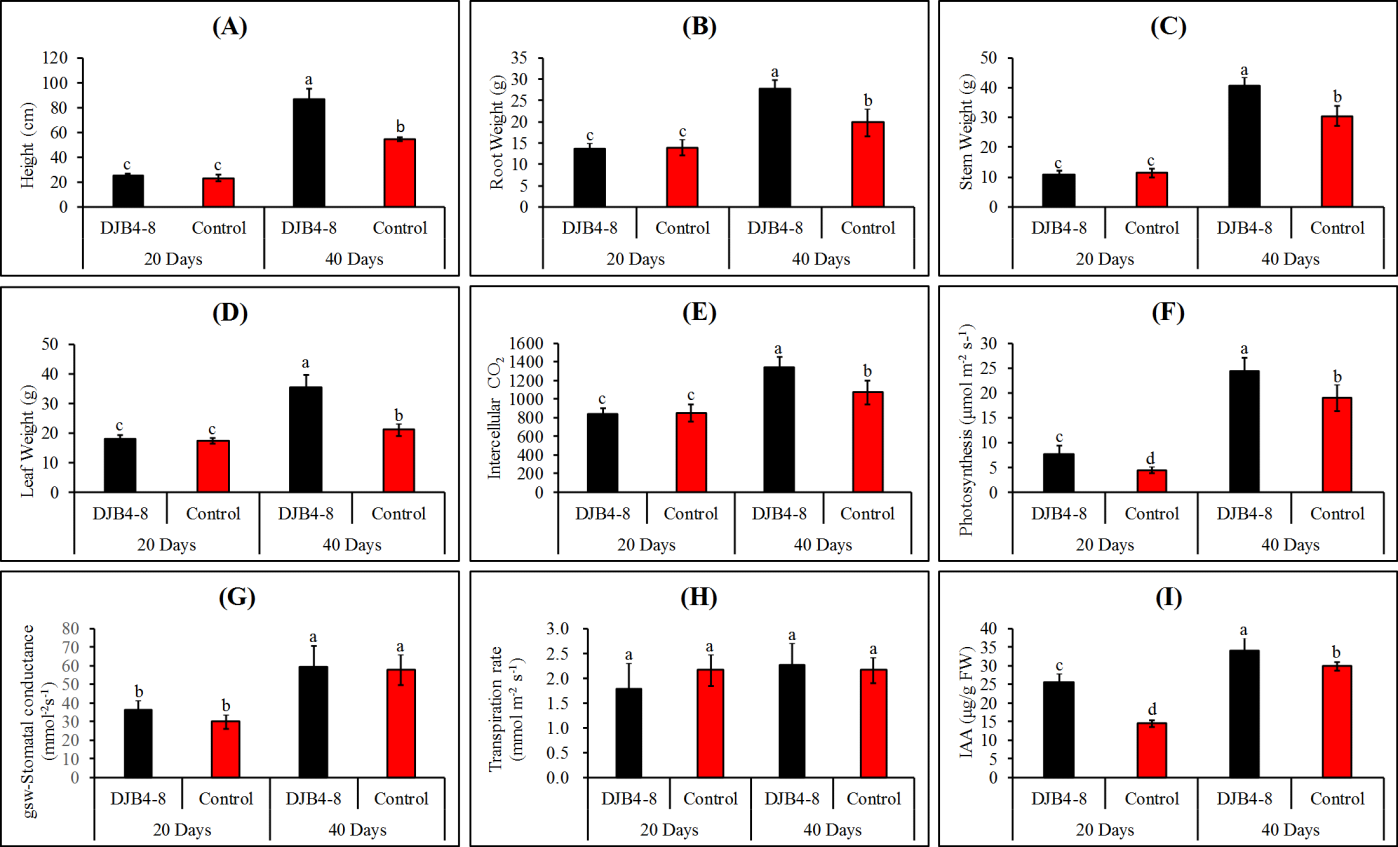
Determination of photosynthetic physiological indexes and IAA content of maize Zhengdan 958 inoculated with strain DJB4–8 at 20 and 40 days. **(A)** Height. **(B)** Root weight. **(C)** Stem weight. **(D)** Leaf weight. **(E)** Intercellular CO_2_. **(F)** Photosynthesis. **(G)** Stomatal conductance (gsw). **(H)** Transpiration rate. **(I)** IAA content. IAA, indole-3-acetic acid. Different letters on the column indicate significant difference at 0.05 level. Student T-test was used for the determination of significant differences.

Strain DJB4–8 considerably enhanced the growth of maize following inoculation of maize Zhengdan 958 (**Figure 5**). The fresh weight of the roots, stems, and leaves and maize heights were assessed for 20 and 40 days following inoculation with strain DJB4-8. According to the results, inoculation treatment could significantly improve maize agronomic indicators. There was no significant difference in various agronomic indicators of maize at 20 days compared to the control group without inoculation, but significant differences were noted at 40 days (**Figures 6A–D**). Other physiological markers, such as intercellular CO_2_, stomatal conductance, and transpiration, did not significantly change 20 days after inoculation with maize strain DJB4-8, with the exception of a notable increase in photosynthesis. Nonetheless, intercellular CO_2_ and photosynthesis in maize increased dramatically 40 days after inoculation, although transpiration and stomatal conductance did not alter appreciably (**Figures 6E–H**). In maize Zhengdan 958 inoculated with strain DJB4-8, root growth hormone (IAA) was detected at 20 and 40 days. The results indicated that the inoculation of strain DJB4–8 significantly increased IAA in maize at both times, reaching 3.62 μg/g FW at 40 days (**Figure 6I**). The examination of the growth characteristics of maize Zhengdan 958 inoculated with strain DJB4–8 shows that inoculation with PSB strain DJB4–8 can greatly enhance physiological and biochemical markers and boost growth at 40 days.

### Overall evaluation of transcriptome data and gene function annotation

A total of six maize root samples of maize Zhengdan 958 were sequenced at 40 days following the inoculation with PSB strain DJB4-8. The average comparison rate with maize reference genome B73_v4 was 80.46%, and the average percentages of sequencing quality indicators Q20 and Q30 were 97.89% and 93.63%, respectively, indicating that the quality of sequencing data was high (**Supplementary Table S5**), and the length distribution information of transcripts is shown in **Supplementary Figure S1**. The NCBI Sequence Read Archive (SRA) database received the original transcriptome sequencing data, and the accession number PRJNA1118104 was acquired. There were 22,750 genes in the inoculated treatment group and control group, accounting for 89.89% of the total number of genes, while 1,085 and 1,473 genes were unique to the inoculated treatment group and the control group, respectively (**Supplementary Figure S2A**). In addition, thermogram analysis showed that the samples in the same group were highly similar, indicating that the transcriptome sequencing data were highly repeatable (**Supplementary Figure S2B**). All genes were annotated in the public database, and the number of gene annotations in each database was obtained (**Supplementary Figure S3**).

### Functional enrichment analysis of DEGs in maize inoculated with strain DJB4-8

**Figure 7:**
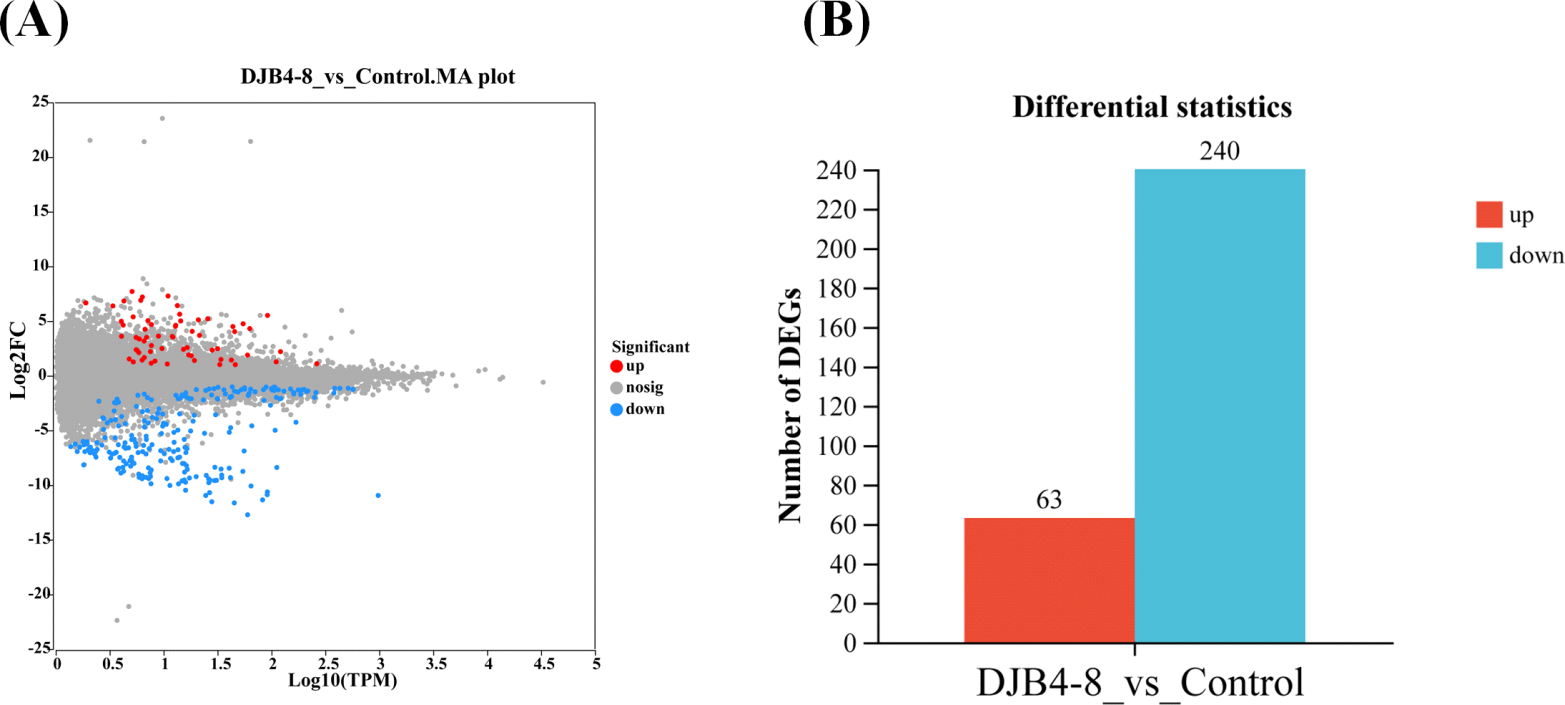
The DEGs in the root transcriptome of maize Zhengdan 958 inoculated with strain DJB4-8. **(A)** The Minus-versus-Add (MA) plot of gene transcriptome expressed genes. **(B)** The number of DEGs (bar). DEGs, differentially expressed genes.

The DEGs between the inoculated and the control groups were screened to examine the changes in gene expression brought about by the DJB4–8 inoculation of maize. This investigation identified 303 DEGs, of which 240 were downregulated and 63 were upregulated (**Figure 7**). Randomly selected 12 important genes with high and low expression in DEGs were chosen for expression verification using qRT-PCR. According to the findings, nine genes’ expression trends aligned with RNA-seq. However, the expression trends of three genes (Zm00001eb382310, Zm00001eb264870, and Zm00001eb093660) did not align with RNA-seq (**Supplementary Figure S4**). Overall, the results of transcriptome sequencing were relatively reliable.

**Figure 8:**
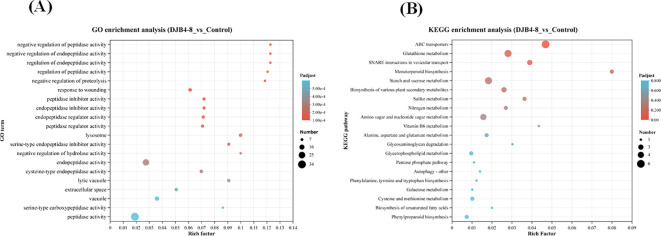
Functional enrichment analysis of maize transcriptome responses to DJB4–8 colonization. **(A)** Enrichment of DEGs in GO database. **(B)** Enrichment of DEGs in KEGG database. Note: The vertical axis represents the GO term/pathway name, and the horizontal axis represents the rich factor—the more significant the rich factor, the greater the degree of enrichment. The dot’s size shows the number of genes in this GO term/pathway, and the dot’s color reflects the various *p*-adjust ranges. DEGs, differentially expressed genes; KEGG, Kyoto Encyclopedia of Genes and Genomes.

**Figure 9:**
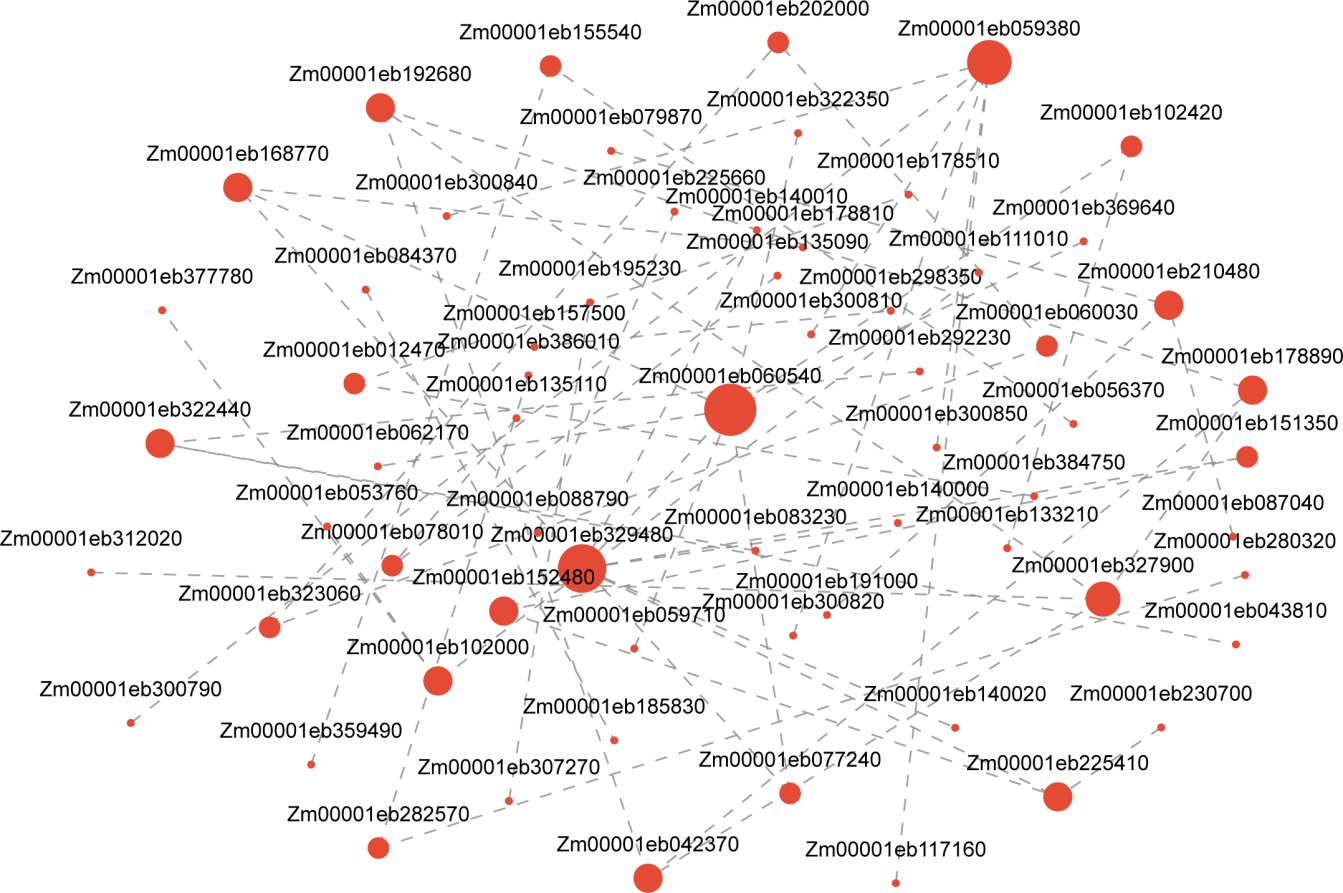
Analysis of protein network regulation predicted by DEGs. Note: Nodes represent genes, and edges represent interactions between two genes. The size of a node is proportional to its connectivity. DEGs, differentially expressed genes.

Using the GO database to annotate DEGs and conducting enrichment analysis separately, it was found that DEGs were mainly enriched in hydrolase activity, oxidoreductase activity, serine hydrolase activity, signaling receptor activity, carbohydrate phosphatase activity, nutrient reservoir activity, and glutathione transferase activity in terms of aspects of biological function. Biological processes (BPs) are mainly enriched in items such as response to stimulus, proteolysis, and defense response. Cellular components (CCs) are mainly enriched in the plasma membrane, extracellular region, etc. (**Figure 8A**). After annotating DEGs and conducting separate enrichment analyses using the KEGG database, it was discovered that DEGs were primarily enriched in metabolic pathways like glutathione metabolism (map00480), starch and sucrose metabolism (map00500), amino sugar and nucleotide sugar metabolism (map00520), and plant hormone signal transduction (map04075) (**Figure 8B**). The analysis of the predicted protein interaction network based on DEGs showed that genes such as trehalose biosynthesis process (Zm00001eb327900), cytoskeleton (Zm00001eb155540), xylan acetylation (Zm00001eb329480), synthesis (Zm00001 eb059380), and trehalose metabolism in response to stress (Zm00001eb 178890) play a decisive role in protein network interactions (**Figure 9**).

### Metabolite identification and DAM analysis of maize inoculated with strain DJB4-8

In the metabolome data of the interaction between maize and strain DJB4-8, 3,069 and 5,740 metabolic ions were identified in cationic and anionic modes, respectively. Ultimately, 1,104 and 1,029 metabolites were identified, with 555 and 467 metabolites annotated to the KEGG database, respectively (**Supplementary Table S6**). PLS-DA indicates that under both anionic and cationic conditions, the greater the sample separation between the inoculation treatment group and the control group, the more significant the classification effect (**Supplementary Figure S5**). Meanwhile, the interpretability of components undefined d1 and two under cations was 23.8% and 16.2% (**Supplementary Figure S5A**), while under anions, it was 25.4% and 20.6% (**Supplementary Figure S5B**), respectively.

**Figure 10:**
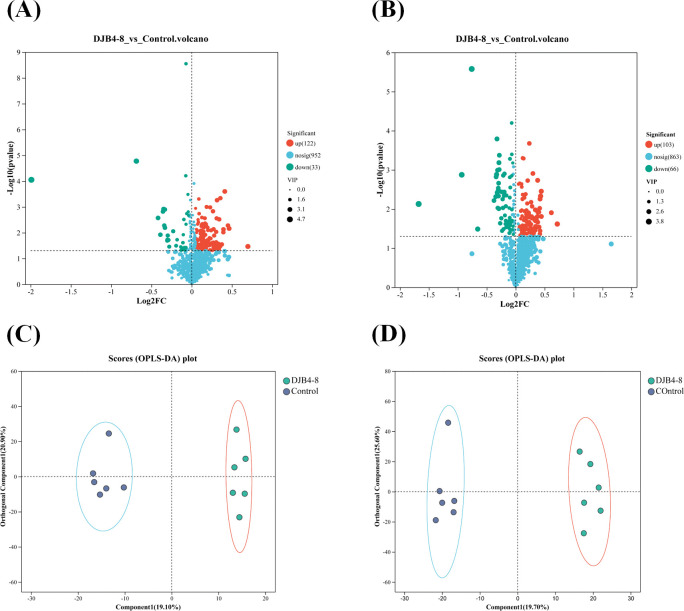
The DAMs in the root metabolome of maize Zhengdan 958 inoculated with strain DJB4-8. **(A)** DAMs in the metabolome under cationic conditions. **(B)** DAMs in the metabolome under anionic conditions. **(C)** OPLS-DA comparative analysis of metabolites under cationic conditions. **(D)** OPLS-DA comparative analysis of metabolites under anionic conditions. DAMs, differentially accumulated metabolites; OPLS-DA, orthogonal partial least squares–discriminant analysis.

Orthogonal partial least squares–discriminant analysis (OPLS-DA) was utilized with univariate multiple analysis and t-test to screen for DAMs and perform significant difference analysis on the identified metabolic characteristics. Out of 115 DAMs screened in cationic conditions, 122 showed upregulation, and 33 showed downregulation (**Figure 10A**). After 169 DAMs were screened in anionic conditions, 103 showed upregulation, and 66 showed downregulation (**Figure 10B**). The OPLS-DA model analysis indicated significant differences between the groups. It revealed that the first predicted principal component release rate and the first orthogonal component release rate under cation were 19.10% and 20.90%, respectively (**Figure 10C**), while under anion, they were 19.70% and 25.60%, respectively (**Figure 10D**).

### Metabolite enrichment analysis of metabolic differences in the interaction between maize and strain DJB4-8

**Figure 11:**
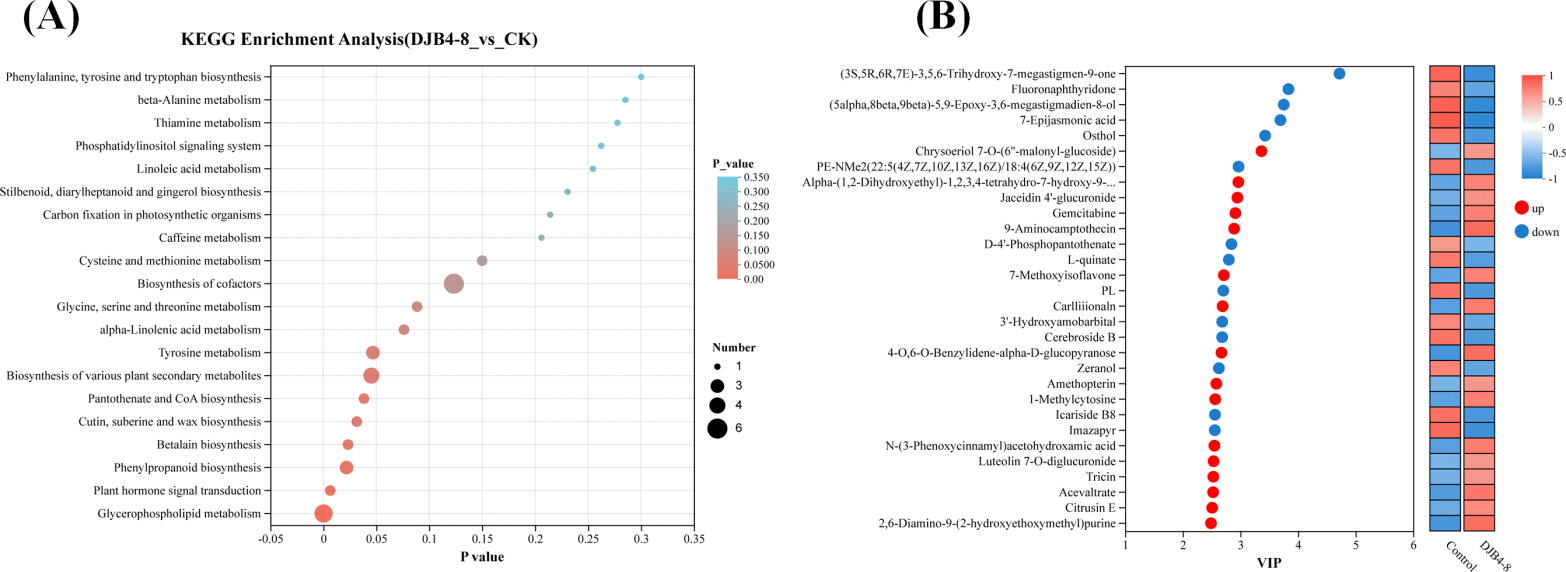
Enrichment of DAMs after strain DJB4–8 inoculated into maize. **(A)** Bubble diagram of DAMs enrichment in the KEGG database. **(B)** VIP analysis of metabolites. Note: In panel A, the abscissa denotes the enrichment ratio (metabolite set enrichment analysis score), while the ordinate specifies annotated KEGG pathways. Bubble diameter is proportional to metabolite set cardinality per pathway. Color gradient corresponds to the statistical significance level (−log10-transformed FDR-adjusted *p*-values). In panel B, the left ordinate displays metabolites ranked by variable importance in projection (VIP) scores derived from OPLS-DA modeling, with bubble size reflecting VIP magnitude. The right panel presents a hierarchical clustering heatmap of z-score normalized relative abundance, where columns represent experimental replicates, and rows indicate significantly regulated metabolites (VIP > 1.0, *q*-value < 0.05). DAMs, differentially accumulated metabolites; KEGG, Kyoto Encyclopedia of Genes and Genomes; FDR, false discovery rate; OPLS-DA, orthogonal partial least squares–discriminant analysis.

Through KEGG pathway enrichment analysis of DAMs, the key metabolic pathways of maize-responsive strain DJB4–8 were further identified (**Figure 11**). The top 20 pathways with the highest enrichment level (lowest *p*-value) were selected from the KEGG database, such as biosynthesis of cofactors (map01240), glycerophospholipid metabolism (map00564), biosynthesis of various plant secondary metabolites (map00999), phenylpropanoid biosynthesis (map00940), and tyrosine metabolism (map00350), which are the metabolic pathways with higher enrichment levels (**Figure 11A**), indicating that metabolites in these pathways may play important roles in the interaction between maize and strain DJB4-8. Furthermore, clustering heatmaps and variable importance in projection analysis have shown that metabolites such as 2,6-diamino-9-(2-hydroxyethoxymethyl)purine, acevaltrate, and tricin are the main upregulated metabolites in DAMs, while (3*S*,5*R*,6*R*,7*E*)-3,5,6-trihydroxy-7-megastigmen-9-one, Fluoronaphthyridone, and others are the main downregulated metabolites in DAMs (**Figure 11B**).

### Combined transcriptome and metabolome analysis of the interaction between maize and strain DJB4-8

**Figure 12:**
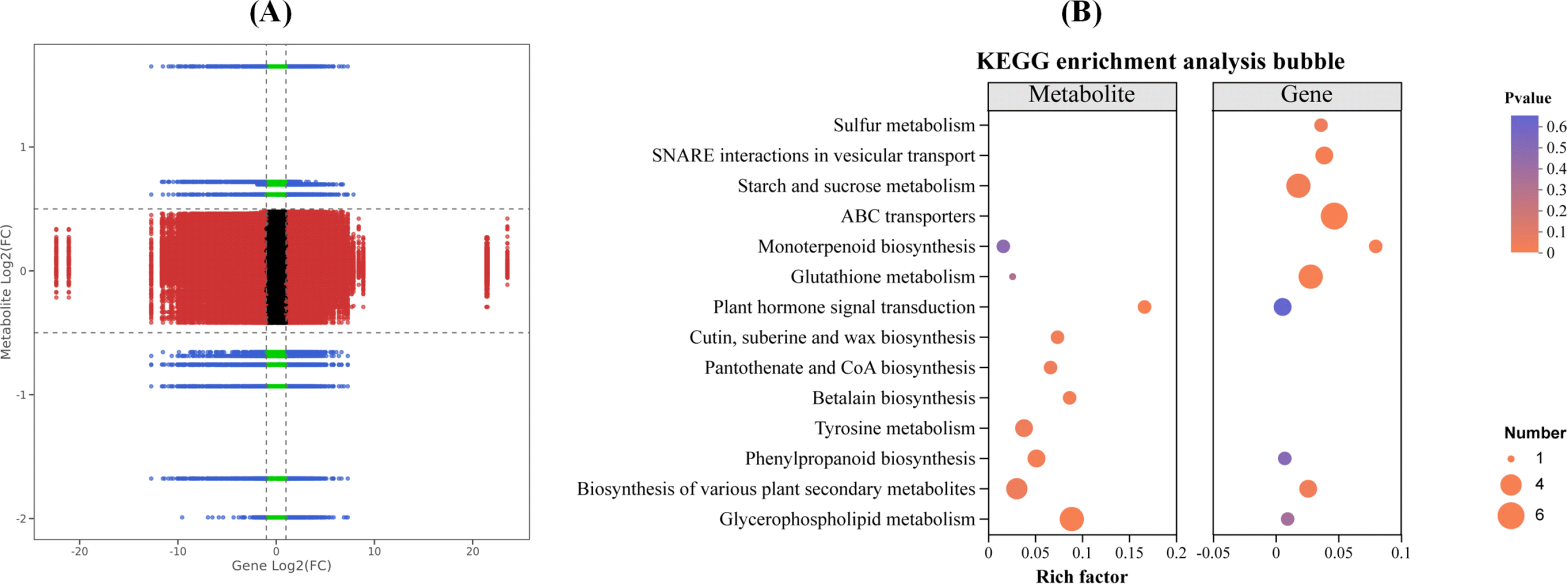
Combined analysis of transgenic and metabolome data after inoculation of strain DJB4–8 in maize. **(A)** Nine-quadrant plot of transcriptome and metabolome expression correlation. **(B)** KEGG enrichment bubble plot of transcriptome and metabolome. KEGG, Kyoto Encyclopedia of Genes and Genomes.

The combined analysis of transcriptomics and metabolomics of the interaction between maize and strain DJB4–8 simultaneously locates DEGs and DAMs on the KEGG pathway map to better evaluate the relationship between genes and metabolites. The nine-quadrant plot analysis indicates a correlation between the transcriptome and metabolome data (**Figure 12A**). The response strain DJB4–8 of maize Zhengdan 958 has 23 and 34 transcriptome- and metabolome-associated genes and differential substances, respectively; KEGG has enriched multiple metabolic pathways, such as glutathione metabolism, starch and sucrose metabolism, biosynthesis of various plant secondary metabolites, amino sugar and nucleotide sugar metabolism, plant hormone signal transduction, and pentose phosphate pathway (**Figure 12B**). These metabolic pathways play important roles in various stages of plant growth.

## Discussion

Scientific researchers have always focused on improving crop yield and quality in agricultural production. With the deepening of microbiology research, using microbial resources to promote crop growth and enhance stress resistance has achieved rich research results in recent years (Guzmán et al., 2025; Sengupta and Gunri, 2015; Gupta et al., 2022; Adeleke and Babalola, 2021). As an important kind of PGPR, PSB can improve the content of available phosphorus in soil by dissolving insoluble phosphate in soil and then promoting crop growth (Panhwar et al., 2011; Ahemad, 2015). This study isolated 41 bacterial strains from the rhizosphere soil of four maize varieties. *B. gladioli* DJB4–8 was evaluated through *in vitro* analysis of plant growth-promoting characteristics and 16S rRNA molecular identification. Strain DJB4–8 exhibited the highest activity-promoting activity of any strain *in vitro*, and it could solubilize phosphate at a level of 8.99 mg/L, much greater than that of other strains. This outcome demonstrates the potential for *B. gladioli* DJB4–8 to be a superior PGPR. Similar to our study, Pande et al. (2017) isolated a PSB strain from the maize rhizosphere that exhibited potent *in vitro* PGP characteristics. Many PSB strains, such as *Pseudomonas aeruginosa*, *Enterobacter aerogenes*, and *Stenotrophomonas maltophilia*, have been screened (Panhwar et al., 2012; Sadiq et al., 2013; Amri et al., 2023). This greatly enriches the PGPR resource library and provides valuable microbial resources for the application of microorganisms in agricultural production. Compared with the reported PGPR, strain *B. gladioli* DJB4–8 exhibits a unique ecological niche preference. It possesses various PGP traits, including ammonia synthesis and iron excretion, and is capable of proliferating in a medium where ACC serves as the exclusive nitrogen source. Specifically, it possesses a high capacity to solubilize phosphorus (**Table 1**), indicating promise for study and use.

The prerequisite for the symbiotic system formed by PGPR and plants to exert its promoting effect is that microorganisms can successfully colonize plant tissues (Compant et al., 2010). This study integrated high-resolution surface morphology characterization using SEM and monitoring with GFP labeling to reveal the colonization pattern of PSB strain *B. gladioli* DJB4–8 in maize rhizosphere at a three-dimensional spatial scale. Strain DJB4–8 effectively infects the maize root surface and establishes a persistent symbiotic relationship by generating many microcolonies on maize Zhengdan 958 roots, as demonstrated by the SEM images. Certain studies have indicated that the preferential adhesion of bacterial cells to root hair regions and intercellular spaces of the root epidermis may correlate with their chemotactic responses and resilience to stressors, including drought and salinity (Kranawetter and Sumner, 2025; Verma et al., 2021). It may also promote the local enrichment of P through physical adsorption mechanisms (Liu et al., 2019; Sharma and Sharma, 2019). It is worth noting that GFP labeling showed clustering of strain DJB4–8 in the inner layer of maize roots (**Figures 4E**), suggesting that it may enter the plant’s symbiotic system through intercellular hyphae or active transport. It provides a reference for studying cross-border interaction mechanisms in the *Burkholderia* genus. The rhizosphere microenvironment has also been shown to dramatically increase the expression level of the acid phosphatase gene (*phoA*) in the genome of certain PSB strains, improving their capacity to solubilize phosphate (Li et al., 2023b; Liu et al., 2020; Dey et al., 2021). At the same time, some PSB strains carry gene clusters encoding the Type III secretion system in their genomes, which may be involved in host-specific signal recognition (Preston et al., 2001; Stringlis et al., 2019). The symbiotic P release mechanism between strain DJB4–8 and maize still needs further research.

Both photosynthesis and respiration in plants depend on P. The carbon fixation mechanism and the photosynthetic electron transport chain are essential, facilitating the production of ATP and NADPH (Malhotra et al., 2018; Amthor, 2023). Photosynthesis and respiration intensity levels were significantly higher in maize Zhengdan 958 infected with the PSB strain DJB4–8 than in the control in this study. After 40 days of inoculation, it was noticed that maize’s photosynthetic physiological activity changed significantly, possibly related to PSB’s improvement of maize P absorption. This study also found that after inoculation with strain *B. gladioli* DJB4-8, the IAA content in maize roots significantly increased, and the developmental pattern of maize roots was significantly altered considerably, increasing the fresh weight of maize roots. Quantitative analysis by HPLC showed that the IAA concentration secreted by DJB4–8 in the maize rhizosphere was as high as 34.03 mg/L, which was 1.14 times higher than that in the control group without inoculation (*p* < 0.05). Some scholars have detected high concentrations of IAA and tryptophan (Trp) in plant rhizosphere through targeted metabolomics. Combined with key genes for IAA synthesis annotated in the strain genome, such as *ipdC* and *aldH*, it has been confirmed that PGPR can synthesize IAA through dual channels of the indole-3-acetamide pathway and tryptophan-dependent pathway (Luziatelli et al., 2020; Li et al., 2018). This multi-pathway synergistic IAA synthesis strategy may help plants adapt to different growth environments. In this study, strain DJB4–8 significantly upregulates genes related to tryptophan synthesis in the transcriptome (map00400), which may be associated with PSB stimulation. Some research has found that PGPR stimulate plant tryptophan metabolism toward IAA synthesis by secreting small molecule metabolites such as IAA or inducing reactive oxygen species (ROS) signals (Deng et al., 2024; Mir et al., 2020). Additionally, after plant inoculation with PGPR, some hormones such as gibberellin drive stem elongation, and IAA enhances assimilate transport capacity, increasing aboveground biomass and maintaining growth through efficient phosphorus absorption, exhibiting a carbon–phosphorus synergistic optimization effect (Mogal et al., 2020). The transcriptome results of this study’s interaction between strain DJB4–8 and maize revealed notable alterations in the expression levels of genes linked to the plant hormone signaling pathways in maize. It implies that strain DJB4–8 may impact maize growth and development by controlling the plant hormone signaling pathways.

The shikimic acid pathway produces phenylalanine, the precursor to the phenylpropane metabolic pathway. Several enzymes catalyze the synthesis of various secondary metabolites necessary for plant growth and development. In the phenylpropane metabolic cycle, lignin, the main component of plant cell walls, forms conduits, moves water and mineral components, and provides mechanical support for plants (Muro-Villanueva et al., 2019). It is a leading creator of systems for material transportation and plant support. Furthermore, this system produces flavonoid chemicals such as flavonoids, flavones, and isoflavones, which are essential for plant growth and development and can act as signaling molecules to help generate root nodules (Meng et al., 2022; Tohge et al., 2013; Wu et al., 2024). After inoculation with DJB4-8, key genes (Zm00001eb133780 and Zm00001eb286490) involved in the phenylpropanoid biosynthesis pathway in maize roots were significantly upregulated. This synergistic change suggests that DJB4–8 may indirectly regulate maize growth by regulating the phenylpropanoid pathway.

Zeatin, as one of the most active members of the cytokinin (CK) family, plays a core regulatory role in plant growth and development, stress response, and microbial interactions (Schäfer et al., 2015; Li et al., 2021). Zeatin drives the G1/S phase transition by activating cell cycle proteins (CycD3) to maintain cell activity (Lu et al., 2025). It can also delay leaf senescence by inhibiting the expression of SAG12 (senescence-related gene) while activating the expression of sucrose transporters (SUC2) to promote the transport of assimilates to grains (Wang et al., 2025; Swartzberg et al., 2011). Genes linked to the zeatin biosynthesis pathway in maize showed altered expression levels following inoculation with strain DJB4-8, suggesting that strain DJB4–8 may influence maize cell division and growth by controlling zeatin production. Glutathione is an important antioxidant that can eliminate free radicals in plants and protect cells from oxidative damage (Margis et al., 2008). This study found that after inoculation with strain DJB4-8, the expression levels of genes related to the glutathione metabolism pathway in maize changed, which may be related to the oxidative stress response triggered by the initial colonization of strain DJB4-8.

The pentose phosphate pathway (PPP) is key in plants’ biosynthetic and energy metabolism processes (Kruger and Von Schaewen, 2003). Genes associated with the pentose phosphate system in maize exhibited changed expression levels when strain DJB4–8 was introduced. Strain DJB4–8 may regulate the pentose phosphate pathway, affecting energy metabolism and maize biosynthesis. Genes linked to the PPP, such as G6PD and 6PGD, were markedly elevated during the late contact stage (Barcia-Vieitez and Ramos-Martínez, 2014; Spielbauer et al., 2013). In addition to promoting phenylpropanoid production, the resultant NADPH may affect redox equilibrium by controlling the thioredoxin system (Zhang et al., 2024; Li et al., 2023c). This process of energy coupling across metabolic pathways may serve as a fundamental basis for enhancing host stress resistance by DJB4-8. After inoculation with PSB, the growth and development of maize were promoted. It may be related to the important role of NADPH and intermediates produced by the PPP in biosynthesis. As a reducing agent, NADPH participates in synthesizing biomolecules such as fatty acids, sterols, and nucleic acids, providing the necessary material and energy basis for the growth of maize plants. At the same time, the intermediate products produced by the PPP also provide raw materials for the synthesis of cell wall structural substances, enhancing the mechanical strength and stability of the cell wall and helping to improve the stress resistance of maize plants (Wurtele and Nikolau, 1986; Pugin et al., 1997).

Following the DJB4–8 inoculation of maize, transcriptome DEGs were enriched in KEGG for carbon amino sugar and nucleotide sugar metabolic pathways, which are centered around the *GFAT* and *GNA1* genes, and *UGD* and *UGP* gene clusters contribute to cell wall synthesis, glycosylation modification, and disease defense. Since nucleotide sugar synthesis is a high-energy process, PSB may raise ATP levels by improving phosphorus absorption, blocking the conformational sites of hexokinase (HXK), and encouraging carbon flow into nucleotide sugar metabolism (Reiter and Vanzin, 2001; Seifert, 2004; Bar-Peled and O’Neill, 2011). Research in the recent past has demonstrated that rhizosphere microorganisms can increase UDP glucuronic acid secretion and encourage microbial chemotaxis colonization (Velmourougane et al., 2017). The accumulation of compounds like *N*-acetylglucosamine is advantageous for cell wall synthesis, maintaining cell osmotic balance, and boosting plant stress resistance (Yoo et al., 2021).

Metabolome analysis revealed that distinct metabolites in the interaction between maize and strain DJB4–8 were considerably enhanced in the glycerophospholipid metabolic pathway. This phenomenon suggests complex metabolic interactions between PSB and host plants, which may involve multiple biological processes such as phosphorus activation, cell membrane remodeling, signal transduction, and defense response (Li et al., 2022a). Glycerol phospholipids are the main components of precursors of signaling molecules and cell membranes, such as phosphatidic acid and phosphatidylinositol. Their metabolic changes may reflect the adaptive adjustment of plants to the action of microbial agents (Dedinaite and Campbell, 2000; Green et al., 2019). In the phospholipid metabolism pathway, phospholipid derivatives (phosphatidic acid) participate in the signal transmission of plant hormones (auxin and jasmonic acid). PSB may activate host defense enzymes [superoxide dismutase (SOD), peroxidase (POD), and catalase (CAT)] by secreting hormone analogs such as IAA, thereby regulating the expression of genes related to glycerophospholipid metabolism (Ge and Zhang, 2015). Simultaneously, glycerophospholipid metabolism interacts with channels like glycolysis and the tricarboxylic acid cycle (TCA), and its enrichment may be a result of plants modifying their energy allocation to accommodate the higher phosphorus absorption and utilization efficiency brought about by microbial agents (Li et al., 2023a, 2022b). The metabolic products of glycerophospholipids (phosphatidic acid) may optimize the plant’s response to PSB promotion. These may function as second messengers and mediate the synergistic action of IAA signaling and the antioxidant enzyme system (Hou et al., 2024). This study indicates that strain DJB4–8 can promote maize root development by secreting IAA, which may enhance phosphorus absorption capacity, increase oxidative stress kinase activity during the maize seedling stage, reduce oxidative damage, and accelerate maize growth.

The biosynthesis of various plant secondary metabolites is another important metabolic pathway for enriching differential metabolites in this study’s metabolome. Plant secondary metabolites (phenols, terpenes, and alkaloids) are important chemical mediators for plants to adapt to environmental stress, resist pathogen invasion, and interact with microorganisms (Firáková et al., 2007; Ko et al., 2010). Inoculating corn with PSB strain DJB4-8 may activate the secondary metabolic network of corn by regulating plant defense response, phosphorus utilization efficiency, or microbial host signaling communication. One important tactic that plants use to deal with biotic and abiotic stress is the manufacture of secondary metabolites, which may also contribute to promoting PSB. Some secondary metabolites (citric acid and malic acid) can directly chelate insoluble phosphorus in soil and synergistically improve phosphorus availability with organic acids secreted by PSB (Bai et al., 2024; Xia et al., 2024). In addition, secondary metabolites may optimize phosphorus absorption efficiency by regulating the expression of phosphorus transporters such as the PHT1 family. In the meantime, the activation of secondary metabolic pathways may reflect the redistribution of carbon sources in plants when phosphorus levels are high enough (Victor Roch et al., 2019; Zhao et al., 2024). It would balance stress resistance and growth promotion by directing photosynthetic products toward defense-related metabolism rather than necessary growth.

Tricin is a metabolite upregulated in DAMs discovered in this study (VIP = 2.53). Tricin is a flavonoid compound widely present in grasses. Tricin, as a flavonoid compound, has antioxidant activity and may protect maize cells from oxidative damage by scavenging free radicals or activating antioxidant enzyme systems such as SOD. Tricin may play a role in secondary metabolic processes in maize, like phenylpropanoid metabolism, which encourages the production of additional phenolic or flavonoid compounds—these metabolites, which help maize deal with biological stress, may be insect-resistant or antimicrobial (Santhoshkumar et al., 2025; Nephali et al., 2021).

This study elucidated the theoretical foundation of the interaction between *B. gladioli* DJB4–8 and maize, demonstrating that PSB modulate maize development via hormonal signals, phenylpropanoid production, redox equilibrium, and other mechanisms. In the future, we will focus on analyzing the spatiotemporal heterogeneity of the interaction between PSB and the maize rhizosphere at the single-cell level. Employing molecular marker technology to monitor the expression of pivotal genes dynamically facilitates a paradigm shift in plant–microbe interaction research from mere descriptive phenomena to precise regulation, hence offering theoretical and technical support for sustainable agriculture.

## Conclusion

This research identified *B. gladioli* DJB4-8, a bacterium that promotes plant development and solubilizes phosphate, from the rhizosphere soil of maize. The strain can establish a persistent symbiotic association in maize roots, markedly enhancing agronomic characteristics and physiological photosynthesis while dramatically increasing the amount of the plant hormone IAA in maize roots. Combined transcriptome and metabolome analysis showed that the key genes and metabolites of maize Zhengdan 958 interacting with strain DJB4–8 were mainly enriched in the key metabolic pathways of plant growth. In conclusion, strain DJB4–8 has excellent potential for development and research. The pertinent research findings offer a theoretical framework for examining the interaction mechanism between PSB and maize, establishing a foundation for the application of PSB in maize cultivation.

## Data Availability

The datasets presented in this study can be found in online repositories. The names of the repository/repositories and accession number(s) can be found below: https://www.ncbi.nlm.nih.gov/, PRJNA1118104.
