# Supplementary material for: Comprehensive analysis of the physiological and molecular responses of phosphate-solubilizing bacterium Burkholderia gladioli DJB4–8 in promoting maize growth
Source: Front Plant Sci. 2025 Jun 13;16:1611674. doi: 10.3389/fpls.2025.1611674 (PMC12202476; doi:10.3389/fpls.2025.1611674)
Supplement: Supplementary file 1 [file DataSheet1.docx]

**Table S1** The sampling information of maize rhizosphere soil

| Maize Varieties | Location |
| --- | --- |
| Zhengdan 958 | 100°22'43″ E, 38°55'50″N |
| Xianyu 335 | 100°26'25″ E, 39°00'07″ N |
| Jingke 968 | 100°26'22″ E, 39°02'08″ N |
| Zhongdan 909 | 100°23'35″ E, 38°53'15″ N |

**Table S2** The primer of 16S rRNA gene and PCR reaction conditions

| Gene/Primer | Reaction component | PCR condition |
| --- | --- | --- |
| 16S rRNA | Taq Master Mix (10 mM) 12.5 µL | Initial Denaturation (95 °C for 5 min), Cycle times (30 cycles), Denaturation (94℃ for 1 min), Annealing (55 °C for 1 min), Annealing (72 °C for 5 min). |
|  | Primer-F (10 mM) 1.0 µL |  |
|  | Primer-R (10 mM) 1.0 µL |  |
|  | DNA 1.0 µL; ddH_2_O 9.5 µL |  |

Primer 27F: AGAGTTTGATCCTGGCTCAG

1492R: AAGGAGGTGATCCAGCCGCA

**Table S3** The standard curve of plant endogenous hormone IAA

| Endogenous hormones | Standard curve | Retention time（min） |
| --- | --- | --- |
| IAA | Y=7.2242x-0.0788; R2=0.9997 | 18.36 |

**Table S4** The primer of qRT-PCR for validation of transcriptome sequencing

| Gen ID | Primer | |
| --- | --- | --- |
|  | Forward primer (F) | Reverse primer (R) |
| Zm00001eb060540 | GAGGGTAGGATCCAGTGCCCT | GAGGGTAGGATCCAGTGCCCT |
| Zm00001eb369640 | CTGGTTCTTCAACCACGAC | CCATGTCGGTTACTGGAATCT |
| Zm00001eb382310 | CGGACATACATGCTTGGAAA | CGGACATACATGCTTGGAAA |
| Zm00001eb002800 | CAGAGGGAGCGAGGCATGGA | CAGAGGGAGCGAGGCATGGA |
| Zm00001eb223380 | GACGACGCGTTCTACCAGAA | GTACATGCATAGCATTAGACG |
| Zm00001eb264870 | GACGACGCCGGCGAGGTCAG | GCATCTGCACGCAGTACTTGC |
| Zm00001eb229150 | GGCGGCGCTTGCTTTGACT | CGTAGCCGAGTTGCAGCCGC |
| Zm00001eb381350 | GACGTCACCGTAGGTGGG | CAGACGTCCGTCGAGCAG |
| Zm00001eb300840 | CAAGACCTTCAAAGTTGACGA | CGTAGCCGAGTTGCAGCCGC |
| Zm00001eb093660 | CAGACAGCATCCTCATCACC | GCATGCAAAAGTCTTCTTGCT |
| Zm00001eb285920 | GCCGTCGCCGCGCCTGTATGC | GGCAGGCGTTGTTGAGCTGCT |
| Zm00001eb315260 | CACACGACGAGTCGGCCTCC | **TGTTTGGAATTATAATCTGCTC** |

**Table S5** Transcriptome sequencing data and sequence alignment information of maize after inoculation with strain DJB4-8

| **Sample** | **Raw reads** | **Clean reads** | **Clean bases** | **Q20 (%)** | **Q30 (%)** | **GC content (%)** | **Total reads** | **Total mapped** |
| --- | --- | --- | --- | --- | --- | --- | --- | --- |
| Control 1 | 41820506 | 41301380 | 6131955891 | 97.87 | 93.57 | 49.71 | 41301380 | 35195128 (85.22%) |
| Control 2 | 43985112 | 43545570 | 6382377178 | 97.93 | 93.75 | 48.45 | 43545570 | 32193497 (73.93%) |
| Control 3 | 43398516 | 42960278 | 6322863493 | 97.91 | 93.69 | 49.56 | 42960278 | 34443710 (80.18%) |
| DJB4-8-1 | 49687798 | 49164224 | 7326744603 | 97.88 | 93.59 | 49.47 | 49164224 | 39351838 (80.04%) |
| DJB4-8-2 | 42173936 | 41768398 | 6247696516 | 97.85 | 93.52 | 49.08 | 41768398 | 33627928 (80.51%) |
| DJB4-8-3 | 43026250 | 42582310 | 6317037216 | 97.92 | 93.68 | 49.51 | 42582310 | 36559979 (85.86%) |

**Table S6** Total ion counts and identification statistics in metabolomics

| **Ion mode** | **All peaks** | **Identified metabolites** | **Metabolites in Library** | **Metabolites in KEGG** |
| --- | --- | --- | --- | --- |
| pos | 3609 | 1104 | 982 | 555 |
| neg | 5740 | 1029 | 983 | 467 |


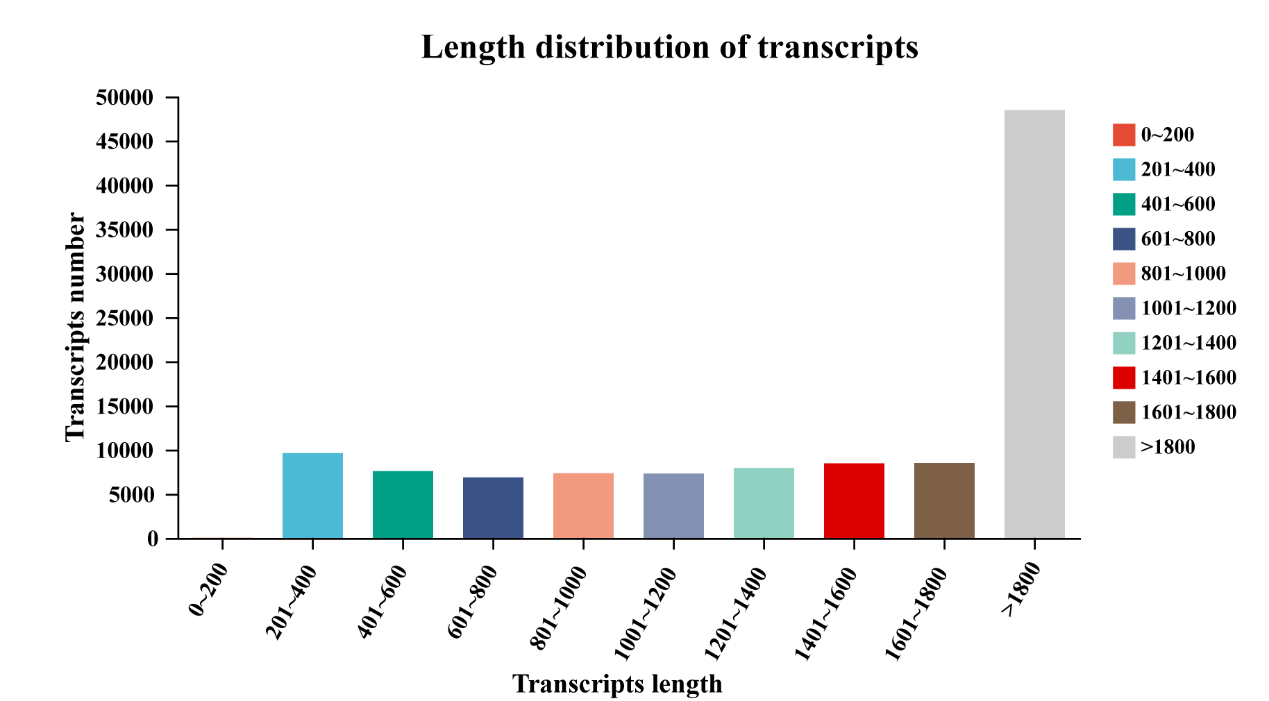


**Figure S1** Transcript length distribution of transcriptome sequencing





**Figure S2** Comparative analysis among transcriptome sequencing samples. (A) Venn analysis of gene distribution between maize inoculated with strain DJB4-8 and uninoculated control group. (B) Correlation heat map of different samples of maize transcriptome sequencing


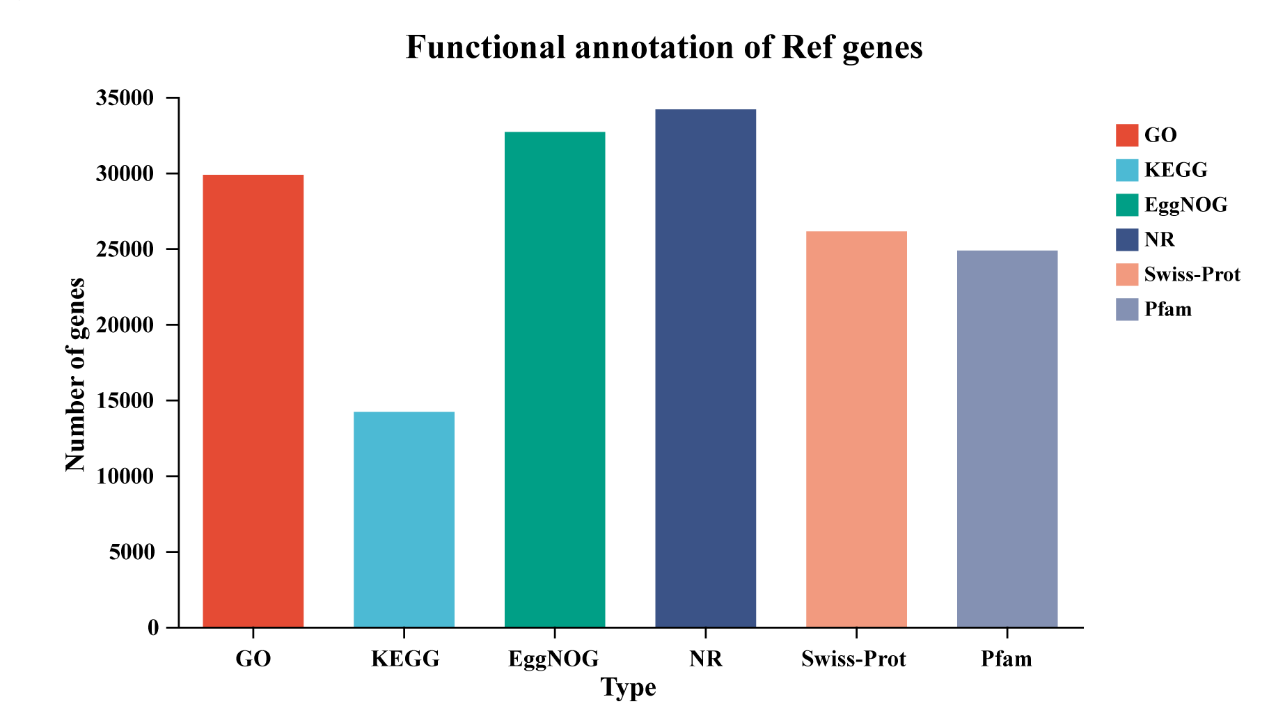


**Figure S3** Annotation of maize transcriptome genes in public databases


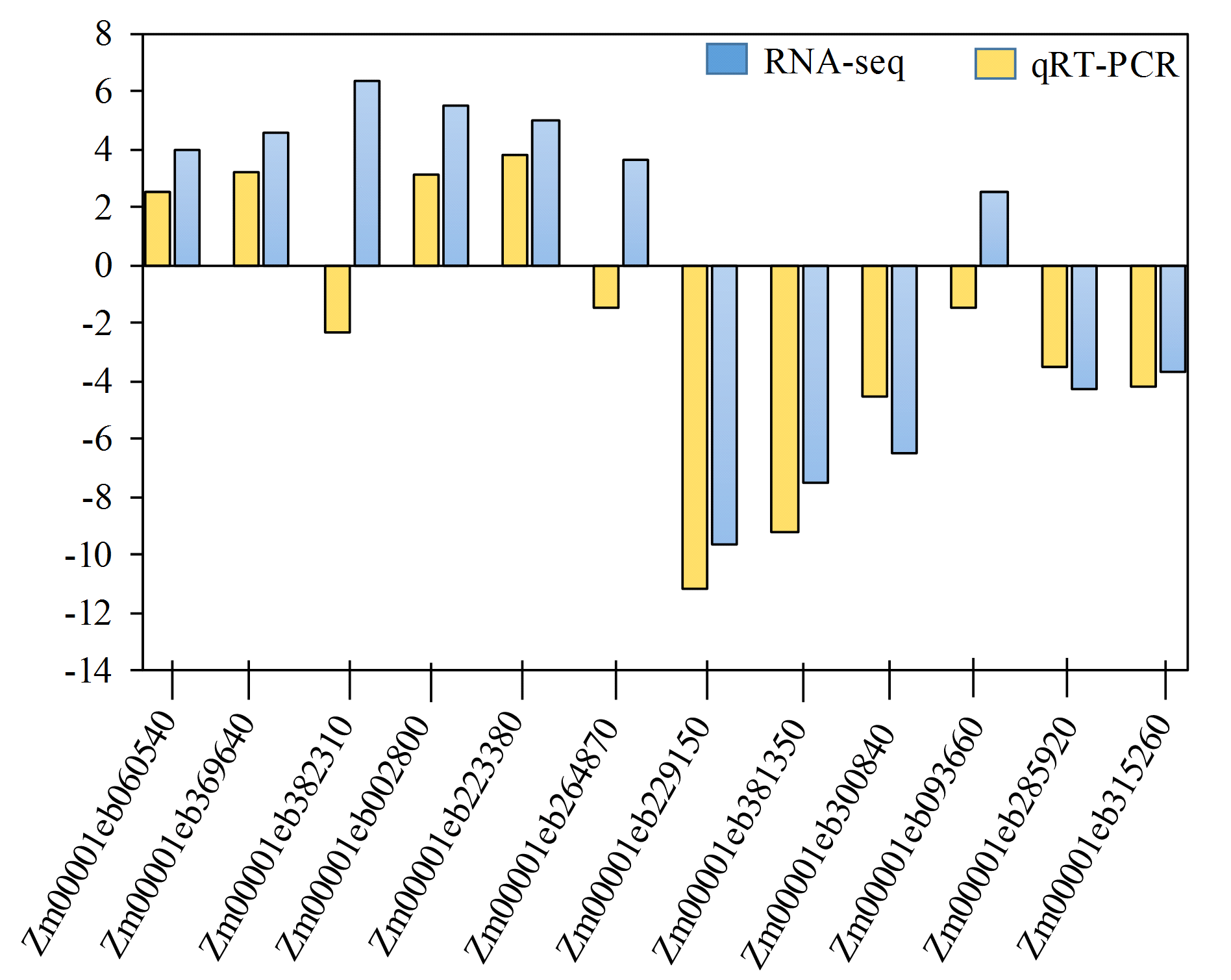


**Figure S4** The qRT-PCR validation of transcriptome sequencing





**Figure S5** Comparative analysis of maize metabolome samples. (A) The PLS-DA analysis under cationic conditions. (B) The PLS-DA analysis under anionic conditions.
